# Evaluation of Cellular Responses of Heterotrophic *Escherichia coli* Cultured with Autotrophic *Chlamydomonas reinhardtii* as a Nutrient Source by Analyses Based on Microbiology and Transcriptome

**DOI:** 10.3390/microorganisms12030452

**Published:** 2024-02-23

**Authors:** Akihito Nakanishi, Natsumi Omino, Tomoyo Nakamura, Saki Goto, Riri Matsumoto, Misaki Yomogita, Naoki Narisawa, Manami Kimijima, Kohei Iritani

**Affiliations:** 1 School of Bioscience and Biotechnology, Tokyo University of Technology, Hachioji 192-0982, Japan; b0a20043f8@edu.teu.ac.jp (N.O.); nakamuraty@stf.teu.ac.jp (T.N.); gotohsk@stf.teu.ac.jp (S.G.); b011929061@edu.teu.ac.jp (R.M.); 2 Graduate School of Bionics, Tokyo University of Technology, Hachioji 192-0982, Japan; g112204255@edu.teu.ac.jp; 3 Bioresource Utilization Sciences, Nihon University Graduate School of Bioresource Sciences, Fujisawa 252-0880, Japan; narisawa.naoki@nihon-u.ac.jp (N.N.); brma22502@g.nihon-u.ac.jp (M.K.); 4 Department of Applied Chemistry, School of Engineering, Tokyo University of Technology, Hachioji 192-0982, Japan; 5 Research Center for Advanced Lignin-Based Materials, Tokyo University of Technology, Hachioji 192-0982, Japan

**Keywords:** *Escherichia coli*, *Chlamydomonas reinhardtii*, cellular response, transcriptomics

## Abstract

Heterotrophic microorganism *Escherichia coli* LS5218 was cultured with flesh green alga *Chlamydomonas reinhardtii* C-9: NIES-2235 as a nutrient supplier. In order to evaluate the cell response of *Escherichia coli* with *Chlamydomonas reinhardtii*, *Escherichia coli* was evaluated with microbial methods and comprehensive gene transcriptional analyses. *Escherichia coli* with *Chlamydomonas reinhardtii* showed a specific growth rate (*µ*_max_) of 1.04 ± 0.27, which was similar to that for cells growing in Luria–Bertani medium (*µ*_max_ = 1.20 ± 0.40 h^−1^). Furthermore, comparing the cellular responses of *Escherichia coli* in a green-algae-containing medium with those in the Luria–Bertani medium, transcriptomic analysis showed that *Escherichia coli* upregulated gene transcription levels related to glycolysis, 5-phospho-d-ribosyl-1-diphosphate, and lipid synthesis; on the other hand, it decreased the levels related to lipid degradation. In particular, the transcription levels were increased by 103.7 times on *pgm* (*p* * < 0.05 (*p* = 0.015)) in glycolysis, and decreased by 0.247 times on *fadE* (*p* * < 0.05 (*p* = 0.041)) in lipolysis. These genes are unique and could regulate the direction of metabolism; these responses possibly indicate carbon source assimilation as a cellular response in *Escherichia coli*. This paper is the first report to clarify that *Escherichia coli*, a substance-producing strain, directly uses *Chlamydomonas reinhardtii* as a nutrient supplier by evaluation of the cellular responses analyzed with microbial methods and transcriptome analysis.

## 1. Introduction

Since ancient times, humans have produced various foods, beverages and pharmaceutical substances with microorganisms, such as sake and bread under alcoholic fermentation by *Saccharomyces cerevisiae* [1], yogurt under lactic acid fermentation by *Lactobacillus delbrueckii* and *Streptococcus thermophilus* [2], pickles under sugar fermentation by *Lactobacillus brevis* [3], seasonings under amino acid fermentation by *Corynebacterium glutamicum* [4], and antibiotics due to the antibacterial action of *Streptomyces griseus* [5]. Particularly, in recent years, *Escherichia coli* has been widely used among industrial microorganisms with advances in genetic engineering techniques such as genetic recombination [6] and genome editing [7], resulting in the recombinant *E. coli* producing 30% of therapeutic proteins in the total production [8] and also producing high amounts of specific substances by controlling their metabolism [9]. Therefore, the use of industrial microorganisms in the production of these substances has been gaining momentum. These industrial microorganisms mainly depend on the supply of nutrients from the outside as heterotrophic microorganisms, and in particular, those strains rely on external supplies of carbohydrates such as glucose [10] and alcohols such as glycerol [11] and ethanol [12] as carbon sources, as well as amino acids such as glutamate [13]. The supply of these nutrients generally requires separation and purification, as well as the construction of facilities and operational systems for sufficient management to avoid contamination with other organisms [14], resulting in a great deal of economic costs and human resources. Therefore, the construction of a system in which industrial microorganisms such as *E. coli* can directly receive nutritional sources such as carbon sources from producers would lead to reducing those costs, which would be of industrial significance.

All organic carbon sources used by industrial microorganisms originate from CO_2_ through photosynthesis. CO_2_ exists in the atmosphere at around 400 ppm as of 2023 [15] and is also abundantly obtained from industrial facilities such as factories, making it an easily accessible carbon resource. Increment of the concentration of CO_2_ in the atmosphere is said to play a role in global warming [15]. Therefore, aiming to easily use a versatile carbon source on earth and avoiding further global warming, the development of effective ways to use CO_2_ in the atmosphere is expected. In the biological field, green algae, which include autotrophic microorganisms such as *Chlamydomonas reinhardtii* and *Chlorella vulgaris,* have recently attracted attention [16]. Their carbon fixation capacities are 10~50 times higher than common terrestrial plants in photosynthesis [17], and they efficiently convert CO_2_ into organic materials such as carbohydrates and oils [18] and vitamins [19]. In fact, in nature, green algae as autotrophic microorganisms supply organic matter to heterotrophic organisms [20]. The construction of the designed system in which heterotrophic industrial microorganisms can directly use the organic matter as nutrient sources produced by green algae as a converter from CO_2_ could simplify the cultivation of heterotrophic microorganisms because there is no need to purify the nutrient source, as there is with conventional systems. To date, there have been no detailed reports on biomass production and the cellular responses of heterotrophic industrial microorganisms directly supplying nutrients from green algae, so our demonstration reveals whether green algae could be directly utilized as a nutrient source.

In this study, alive *C. reinhardtii* cells or cellular extracts from *C. reinhardtii* could provide organic matter derived from CO_2_ to sustain the growth of *E. coli*. The growth responses in these cultures and the changes in the transcriptome of *E. coli* were evaluated. The obtained results provide the possibility to produce biomaterials by *E. coli* using *C. reinhardtii* as a nutrient producer. Therefore, this report could be valuable for production engineering.

## 2. Materials and Methods

### 2.1. Obtainment of Green C. reinhardtii Cells

*Chlamydomonas reinhardtii* strain C-9: NIES-2235 was purchased from the National Institute for Environmental Studies (NIES) (Tsukuba-shi, Ibaraki, Japan). For harvest of viable green algal cells, the strain was cultured under the photobioreactor (PBR) as below: volume: 100 mL in a glass vessel; light intensity: 130 µmol photons·m^−2^·s^−1^ with white fluorescent lamps; gas bubbling: 0.8% CO_2_ gas at 0.3 vvm; temperature: room temperature (23 °C). The cultivation medium was Modified Bold’s basal medium (MBBM): 1.5 mM NaNO_3_, 0.22 mM K_2_HPO_4_, 0.3 mM MgSO_4_·7H_2_O, 0.17 mM CaCl_2_·2H_2_O, 0.43 mM KH_2_PO_4_, 0.43 mM NaCl and necessary components described in a previous report as a reference [21]. In pre-cultivation, values of optical density (OD) at 750 nm in broth were monitored with a spectro-photometer U-2900 (Hitachi-Ltd., Chiyoda-ku, Tokyo, Japan), and the values were converted to cell density via appropriate calibration curves for OD_750_ versus cell density (cells∙mL^−1^) and dry cell weight (DCW) (g∙L^−1^). For obtaining lyophilized cells, *C. reinhardtii* C-9: NIES-2235 was prepared for the supply of those intracellular contents by Meravi Co., Ltd. (Souka-shi, Saitama, Japan).

### 2.2. Preparation of Disrupted Cell Extracts from C. reinhardtii Cells

Disrupted cell extracts were harvested by ultrasonic cavitation with an ultrasonic homogenizer Smurt NR-50M operating at 20 kHz with an ultrasonic horn and a 3 mm diameter tip NS-50M-MT3 (Funabashi-shi, Chiba, Japan). In total, 2 g of lyophilized cells was suspended with 20 mL of MBBM in 100 mL of a glass vial on ice, and the suspension was stirred at 300 rotations per minute (rpm) with a magnetic stirring device. In total, 2 cm of the top of the chip was inserted into the stirred cell suspension and then sonication was performed (on: 30 s; off: 30 s, 40 cycles) on ice. The disrupted suspension was collected with 50 mL of polypropylene centrifuge tubes, and then those tubes were centrifuged at 2500× *g* for 3 min at 23 °C. The supernatants were transferred into other 50 mL of polypropylene centrifuge tubes and similarly centrifuged at 2500× *g* for 3 min at 23 °C again. The supernatants were collected and filled with MBBM up to 100 mL. After the processes, the supernatants were transferred into other 50 mL of polypropylene centrifuge tubes, and highly treated with centrifugation at 10,000× *g* for 5 min at 23 °C. The supernatants were stepwisely filtered with filter paper #3 (pore size: 5 µm) (Advantec, Chiyoda-ku, Tokyo, Japan), a stericup quick release HV sterile vacuum filtration system (pore size: 0.45 µm) (Merck Millipore, Danvers, MA, USA) and a stericup quick release HV sterile vacuum filtration system (pore size: 0.22 µm) (Merck Millipore). The filtered solution was used as a 20 g∙L^−1^ stock solution of disrupted cell extracts.

### 2.3. Cultivation of E. coli Cells

*Escherichia coli* LS5218 [22] was cultivated with 160 strokes per minute (spm) at 30 °C in 3 mL of Luria–Bertani (LB) medium as pre-cultivation. The precultured *E. coli* cells were diluted with saline, and transferred to 10 mL of the main culture media described in the following sentence adjusted to 3.0 × 10^4^ cells·mL^−1^ as initial cell density; the main culture media were prepared as two types of MBBM containing of the disrupted cell extracts of *C. reinhardtii* as 0.1~10 g·L^−1^ or *C. reinhardtii* cells as 2 g·L^−1^. In this paper, when *E. coli* was cultured with *C. reinhardtii* cells, the cells did not mean lyophilized cells but the cells directly prepared after culturing in MBBM. After the inoculation, *E. coli* was cultivated with 160 spm at 30 °C as the main cultivation. The cell growth of *E. coli* was evaluated with a colony-forming unit (cfu) on LB agarose plates as alive cell densities. In detail for analyzing cell viability of *E. coli*, 1 mL was collected from 10 mL of the broth containing *E. coli* and stepwisely diluted to 10^1^~10^8^ times with saline. The dilutants were spread on LB agarose plates and incubated at 30 °C to pick alive cells up. The cell numbers obtained by counting the colonies on the LB agarose plate were used for quantifying alive cell densities as cell viabilities (cells·mL^−1^).

### 2.4. Microscope Observation of E. coli Treated with Gram Staining

Appropriate volumes of broths were collected (normally, 1000 µL at 0 h; 10 µL at 6 h~) and centrifuged at 20,400× *g* for 5 min at 23 °C. In the case of taking 1000 µL of broth, 990 µL of the supernatant was discarded and the residue was suspended by pipetting. The suspension was transferred on a glass slide, dried out, and treated by flame fixation. Based on the favor-G set-S kit system (Shimadzu Diagnostics Corporation, Taito-ku, Tokyo, Japan), staining solution A was dropped on the flame-fixed cells and kept for 1 min. The stained cells were washed with distilled water and then rewashed with a decoloring solution. Staining solution B was dropped on the washed cells and kept for 1 min, and the stained cells were washed with distilled water. The Gram-stained cells were observed with an optical microscope OLYMPUS BX43 system equipped with an OLYMPUS DP74 (Olympus Corp., Shinjuku-ku, Tokyo, Japan) camera and with cellSens Standard software 3.2 installed.

### 2.5. Evaluation of Cell-Viability of C. reinhardtii

For analyzing cell viability of *C. reinhardtii*, 180 µL of the broths was mixed with 20 μL of neutral red (red pigment) (Tokyo Kasei Co., Ltd., Chuo-ku, Tokyo, Japan) for 5 min [23]. The neutral red solution (1.5 mg·mL^−1^) was prepared as follows: 1.5 mg of neutral red was dissolved in 1 mL of phosphate-buffered saline (PBS) (pH = 7.4); the solution was filtered with a 0.22 μm nylon syringe filter (Membrane-Solutions, LLC, Auburn, WA, USA). After the staining process, the cells were washed with PBS, and the viability was evaluated by observing the stain-treated cells on a cell counter plate (Fukae Kasei Co., Ltd., Kobe-shi, Hyogo, Japan) under an optical microscope. The life/death of the cells was decided with the criterion: staining/not staining with neutral red on hemocytometer of improved Neubauer (Watson Co., Ltd., Arakawa-ku, Tokyo, Japan). The cell growth curve was drawn with plotted values of alive cell densities (cells∙mL^−1^) obtained by multiplying cell density (cells∙mL^−1^) by cell survival ratio (%). Cell density was obtained by microscopic observing with a Neubauer-improved hemocytometer (Watson Co., Ltd.). Cell survival ratio was shown with a neutral red method described above.

### 2.6. Quantification of Carbohydrate, Protein, Lipid and Organic Acid in Broth

In preparation for the quantification of carbohydrates, proteins and lipids, the supernatant from 2 mL of broth was collected by centrifugation (2500× *g*; 3 min; 23 °C), and the supernatant was treated with 0.22 µL filter as the filtered flowthrough.

Total carbohydrate concentration in a broth was quantified with a colorimetric carbohydrate assay kit (Cell Biolabs, Inc., San Diego, CA, USA) depending on the phenol–sulfuric acid method. The analysis was performed using the filtered flowthrough on a 96-well plate (type no: 1-1601-06) (Violamo-As one, Osaka-shi, Osaka, Japan) as shown below: 30 µL of the filtered flowthrough was mixed with 150 µL of highly concentrated sulfuric acid; the mixture was heated to 90 °C for 15 min and then also put on ice at 2~3 min; the treated mixture was blended well with 30 µL of developing solution containing phenol; the absorbance value at 490 nm of the blended mixture was monitored in each well of a 96-well plate under a strong vibration mode (5 s, strong mixing mode) at 23 °C as room temperature with a 96-well microplate reader SH-1300Lab (Hitachi High-Tech Science Co., Minato-ku, Tokyo, Japan); the absorbance was evaluated as the value reflected the carbohydrate concentration with a glucose-providing standard curve.

Total protein concentration in a broth was quantified with a colorimetric protein assay kit (Bio-Rad Laboratories, Inc., Hercules, CA, USA) based on the Bradford method. The analytical procedure was with the filtered flowthrough on a 96-well plate (type no: 1-1601-06) (Violamo-As one) as follows: 10 µL of the filtered flowthrough was blended with 200 µL of dye reagent prepared by diluting dye reagent concentrate with distilled water as 20%; the mixture was heated to 23 °C for 15 min; the absorbance value at 595 nm of the mixture was collected in each well of a 96-well plate under a strong vibration mode (5 s, strong mixing mode) at 23 °C as room temperature with a 680-model microplate reader (Bio-Rad Laboratories); the absorbance was quantified as the value reflected the protein concentration with an albumin-providing standard curve.

Total lipids in a broth were quantified and qualified by gas chromatography–flame ionization detector method. Total lipids in the filtered flowthrough were methyl esterified with a fatty acid methylation kit (Nacalai Tesque, Kyoto-shi, Kyoto, Japan). The fatty acid methyl esters were identified and quantified with a capillary gas chromatograph GC-2025 (Shimadzu Corp., Kyoto-shi, Kyoto, Japan) equipped with a DB-23 capillary column (60 m, 0.25 mm internal diameter, 0.15 μm film thickness) (Agilent Technologies, Santa Clara, CA, USA). Heptadecanoic acid (Sigma-Aldrich Co., Saint Louis, MO, USA) was used as an internal standard; rapeseed oil (Merck KGaA, Frankfurter Str., Darmstadt, Germany) was used as a quantitative standard. The details regarding the method were described in a previous report [18].

Each organic acid in a broth was quantified and qualified by ion exclusion chromatography and the post-column pH-buffering electric conductivity detection method. In total, 500 µL of the broth was centrifuged at 20,400× *g* for 1 min at 23 °C, and then the supernatant was collected. The supernatant was percolated with a 0.45 µm filter, and 10 µL of the flowthrough was injected into high-performance liquid chromatography system (guard column: SCR-102H (6.0 mm × 50 mm) (Shimadzu); column: two-tandem Shim-pack SCR-102H (8.0 mm × 300 mm) (Shimadzu); column temperature: 40 °C; mobile phase: mobile phase reagent for organic acid analysis system (Shimadzu); flow rate: 0.8 mL∙min^−1^; electrical conductivity detector (polarity: +; cell-temperature: 43 °C)) as the Prominence Organic Acid Analysis System (Shimadzu). The obtained data were analyzed with Shimadzu-LabSolutions (Shimadzu) software 3.8 SP3.

### 2.7. Measurement of Levels of Gene Translation

*E. coli* was cultivated in *C. reinhardtii*-MBBM and LB medium, as mentioned above. The material for transcriptomic analysis was *C. reinhardtii* cells growing in MBBM (*C. reinhardtii*-MBBM) at a density of 2 g of cells per a litter of culture.

Approximately 5 mg of *E. coli* cells cultivated in *C. reinhardtii*-MBBM or LB medium was collected from the broth by centrifugation at 21,500× *g* for 5 min. The collected cells were mixed with 50 µL of QIAzol Lysis Reagent (QIAGEN, Chuo-ku, Tokyo, Japan) and shaken for 5 min. After keeping the shaken samples at 23℃ for 5 min, 10 µL of chloroform was added and placed on ice for 3 min. The treated samples were centrifuged at 21,500× *g* for 15 min at 4 °C; the supernatant was shaken with 25 µL of isopropanol; the mixture was placed at 23 °C for 10 min. The supernatant was discarded after centrifugation at 21,500× *g* for 10 min, and the precipitant was rinsed with 1 mL of 70% ethanol. The rinsed sample was dried with lyophilizer Refrigerated CentriVap Benchtop Vacuum Concentrator (Labconco Corp., Kansas City, MO, USA) and the dried precipitant was dissolved in 10 µL of RNase-free water. The prepared sample as total RNA was used to synthesize complementary DNA (cDNA) using a ReverTra Ace qPCR RT Master Mix with a gDNA Remover (TOYOBO, Osaka-shi, Osaka, Japan). With the cDNA, quantitative PCR (qPCR) was performed with THUNDERBIRD SYBR qPCR Mix (TOYOBO) using Mx qPCR Systems (Agilent Technologies). The average threshold cycle values were evaluated throughout the logarithmic amplification phase and were normalized by the level of *rrsA*. The qPCR primer pairs (Supplemental Table S1) were designed based on the Primer3Plus algorithm (https://dev.primer3plus.com/index.html accessed on 12 September 2023) using information from each predicted gene sequence obtained via the genome information of NCBI. The figures regarding transcription levels were prepared manually without any software.

## 3. Results

### 3.1. Growth of E. coli with Disrupted Cell Extracts from C. reinhardtii

**Figure 1:**
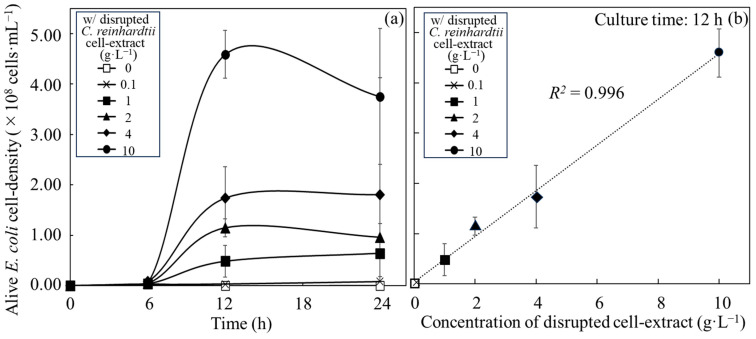
**Growth of *E. coli* depended on concentrations of disrupted cell extracts from *C. reinhardtii.*** Growth of *E. coli* was demonstrated in MBBM and the MBBM containing 0.1, 1, 2, 4 and 10 g∙L^−1^ of disrupted cell extracts from *C. reinhardtii*. As final concentration, 0, 0.1, 1, 2, 4 and 10 g∙L^−1^ of disrupted cell extracts from *C. reinhardtii* were displayed as □, ×, ■, ▲, ◆ and ●. (**a**) Time course profile of alive cell density as a cell growth. (**b**) Correlation curve between the concentration of disrupted cell extract and alive cell density at 12 h cultivation. Error bars indicate the SD of three replicate experiments (n = 3).

The alive cell density of *E. coli* in an autotrophic MBBM with disrupted cell extracts from *C. reinhardtii* was analyzed over time to evaluate whether *E. coli* could use the nutritional sources of the cell extracts from *C. reinhardtii* (Figure 1). *E. coli* was cultivated in MBBM containing disrupted cell extracts adjusted to those concentrations obtained from 0.1–10 g∙L^−1^ of *C. reinhardtii* cells, and the viable cell density was monitored to display the growth over time to evaluate whether *E. coli* can utilize disrupted cell extracts of *C. reinhardtii* as a nutritional source (Figure 1a and Table 1). In the medium without disrupted cell extracts, the alive cell density of *E. coli* reached a maximum cell density of (3.0 ± 0.4) × 10^6^ cfu∙mL^−1^ at 6 h, and the density decreased from 6 h to 12 h. On the other hand, in the medium in which the concentrations of disrupted cell extracts were adjusted to 0.1, 1, 2, 4, and 10 g∙L^−1^, the cell densities of *E. coli* showed maximum or almost maximum cell density of (3.2 ± 0.4) × 10^6^ cfu∙mL^−1^, (4.8 ± 3.2) × 10^7^ cfu∙mL^−1^, (1.2 ± 0.2) × 10^8^ cfu∙mL^−1^, (1.7 ± 0.6) × 10^8^ cfu∙mL^−1^, (4.6 ± 0.5) × 10^8^ cfu∙mL^−1^ at 12 h, and the alive cell density showed a plateau from 12 h to 24 h since each alive cell density at 12 h to 24 h did not show the significant difference. Furthermore, the correlation between the alive cell densities of *E. coli* and the concentration of disrupted cell extracts was evaluated at 12 h of culture when the cell densities had already reached plateaus (Figure 1b). As a result, within the concentration range of disrupted cell extracts defined as 0 to 10 g∙L^−1^, the correlation between the alive cell density of *E. coli* and disrupted cell extracts was shown with a coefficient of determination (*R*^2^) of 0.996, indicating the high correlation.

### 3.2. Comparison of Growth Activities of E. coli with C. reinhardtii Cells and with Disrupted Cell Extracts from C. reinhardtii

**Figure 2:**
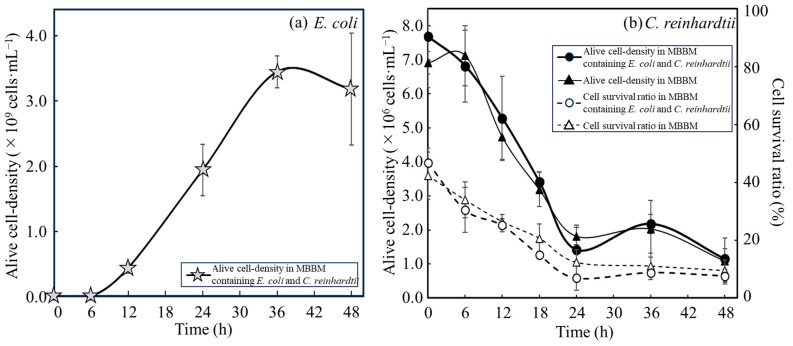
**Growth of *E. coli* and *C. reinhardtii* in the medium containing of *C. reinhardtii* cells.** Growth of *E. coli* and *C. reinhardtii* were shown in MBBM containing *C. reinhardtii* adjusted to 2 g∙L^−1^ as initial concentration. (**a**) Alive cell density of *E. coli* was displayed as growth. Error bars indicate the SD of three replicate experiments (n = 3). (**b**) Alive cell density and cell survival ratio of *C. reinhardtii* were plotted as growth. Closed circle: alive cell density of *C. reinhardtii* with *E. coli* in MBBM; closed triangle: alive cell density of *C. reinhardtii* in MBBM; open circle: cell survival ratio of *C. reinhardtii* with *E. coli* in MBBM; open triangle: cell survival ratio of *C. reinhardtii* in MBBM. Error bars indicate the SD of three replicate experiments (n = 3).

Whether *E. coli* could use *C. reinhardtii* cells as a nutritional source supplier without artificial disruption was evaluated by analyzing the changes in the alive cell density of *E. coli* in MBBM supplied with *C. reinhardtii* cell (Figure 2a and Table 1). As a result of the analyses, the alive cell density of *E. coli* was (4.35 ± 0.77) × 10^8^ cfu∙mL^−1^ in 2 g∙L^−1^ *C. reinhardtii*-MBBM at 12 h. The cell densities showed (1.15 ± 0.17) × 10^8^ cfu∙mL^−1^ and (1.72 ± 0.62) × 10^8^ cfu∙mL^−1^ in 2 g∙L^−1^ and 4 g∙L^−1^ disrupted cell extract-MBBM with significant differences towards that in 2 g∙L^−1^ *C. reinhardtii*-MBBM at 12 h; on the other hand, the density displayed (4.59 ± 0.48) × 10^8^ cfu∙mL^−1^ in 10 g∙L^−1^ disrupted cell extract-MBBM without a significant difference to that in 2 g∙L^−1^ *C. reinhardtii*-MBBM at 12 h. Moreover, the alive cell densities of *E. coli* in 2 g∙L^−1^ *C. reinhardtii*-MBBM at 24 h and 36 h were (19.42 ± 0.39) × 10^8^ cfu∙mL^−1^ and (34.48 ± 2.44) × 10^8^ cfu∙mL^−1^, meaning of the significant increments from 12 h to 36 h. Additionally, the alive cell density of *E. coli* in *C. reinhardtii*-MBBM at 48 h was (31.80 ± 8.56) × 10^8^ cfu∙mL^−1^, indicating a stationary phase after 36 h. As shown above, the proliferations of *E. coli* with either the disrupted cell extracts or the *C. reinhardtii* cells as nutrient sources were proved. However, the specific growth rates of *E. coli* with those were unclear so the rates and doubling time were calculated (Table 1). The growth of *E. coli* in MBBM adjusted to 10 g∙L^−1^ disrupted cell extracts was the best in the medium group adding disrupted cell extracts, and the specific growth rate and the doubling time of *E. coli* from 6 h to 12 h were 0.82 ± 0.27 h^−1^ and 0.90 ± 0.25 h. On the other hand, the specific growth rate and the doubling time of *E. coli* in *C. reinhardtii*-MBBM from 6 h to 12 h were 1.04 ± 0.27 h^−1^ and 0.71 ± 0.22 h, revealing that the growth was higher than that in MBBM adjusted to 10 g∙L^−1^ disrupted cell extracts. In addition, to clarify the viability and proliferation of *C. reinhardtii* in the *C. reinhardtii*-MBBM, the cell survival ratio and the alive cell density of *C. reinhardtii* were analyzed (Figure 2b). As the result of the analysis, the cell survival ratio and the cell density of *C. reinhardtii* in MBBM containing/not containing *E. coli* showed no significant difference, and therefore the alive cell density also displayed no significant difference.

### 3.3. Evaluation of Nutrients Suplied from C. reinhardtii Consumed by E. coli

**Figure 3:**
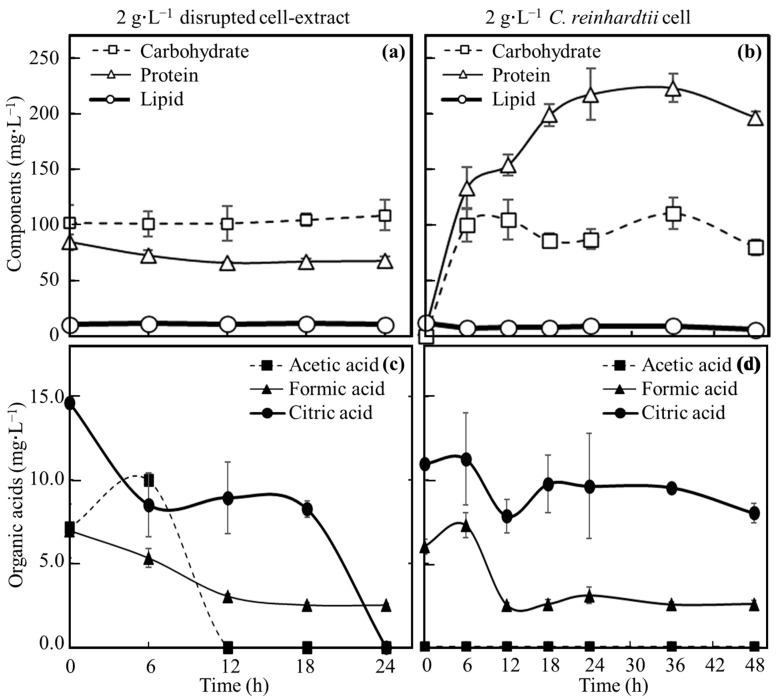
**Time course profile of component concentrations in broths.** Carbohydrate, protein, lipid and organic acids (formic acid, acetic acid and citric acid) in broth added to disrupted cell extracts and cells adjusted to 2 g∙L^−1^ were quantified over time as shown below. Carbohydrates (square), proteins (triangle) and lipids (circle) were shown in disrupted cell extract column (**a**) and in *C. reinhardtii* cell column (**b**). Acetic acid (square), formic acid (triangle) and citric acid (circle) were shown in disrupted cell extract column (**c**) and in *C. reinhardtii* cell column (**d**). Error bars indicate the SD of three replicate experiments (n = 3).

Supernatants of broths derived from 2 g∙L^−1^ disrupted cell extract-MBBM and 2 g∙L^−1^ *C. reinhardtii*-MBBM culturing *E. coli* were analyzed to evaluate each concentration (Figure 3). In 2 g∙L^−1^ disrupted cell extract-MBBM, the concentration of protein was 84.9 ± 7.1 mg∙L^−1^ at 0 h. Thereafter, the concentration of protein decreased to 66.2 ± 1.5 mg∙L^−1^ with the cell proliferation by 12 h, when proliferation stopped. The protein concentrations showed no significant differences observed in 12 h and 24 h. The concentration of carbohydrates was 84.9 ± 7.1 mg∙L^−1^ at 0 h. Although *E. coli* showed growth, the concentration of carbohydrates displayed no significant difference up to 24 h with the growth. Lipid concentrations were 11.1 ± 1.1 mg∙L^−1^ at 0 h and 10.9 ± 0.1 mg∙L^−1^ at 24 h, meaning that the concentration was stable without the increment/decrement. The concentration of formic acid was 7.0 ± 1.6 mg∙L^−1^ at 0 h as the starting point of the culture. The concentration of formic acid decreased to 3.0 ± 0.1 mg∙L^−1^ by 12 h, and afterward, there were no significant changes in the concentrations from 12 h to 24 h. Acetic acid concentration increased from 7.2 ± 0.1 mg∙L^−1^ at 0 h to 10.0 ± 0.6 mg∙L^−1^ at 6 h; however, its presence could no longer be confirmed by 12 h. The concentration of citric acid decreased from 14.6 ± 0.0 mg∙L^−1^ at 0 h to 8.5 ± 1.9 mg∙L^−1^ at 6 h, maintained until 18 h, and interestingly decreased to 24 h, at which point, its existence was no longer confirmed.

In 2 g∙L^−1^ *C. reinhardtii*-MBBM, the protein was not detected at 0 h; however, the protein concentration gradually increased to 198.8 ± 9.7 mg∙L^−1^ at 18 h. Carbohydrate concentration was not measured at 0 h, increased to 100.5 ± 15.1 mg∙L^−1^ in 6 h, and maintained as 80.2 ± 7.3 mg∙L^−1^ at 48 h. Lipid concentrations were 12.3 ± 0.4 mg∙L^−1^ at 0 h and 6.2 ± 4.0 mg∙L^−1^ at 48 h, meaning that the concentration gradually decreased. The formic acid concentration was 6.0 ± 0.5 mg∙L^−1^ at 0 h as the starting point of the culture, and after decreasing to 2.5 ± 0.1 mg∙L^−1^ in 12 h, the concentration was not changed until 48 h. The acetic acid could not be confirmed from the start to the end of the culture. The concentration of citric acid was 11.0 ± 0.0 mg∙L^−1^ at 0 h, and then it decreased to 8.0 ± 0.6 mg∙L^−1^ at 48 h.

### 3.4. Aging Variation in Cell Size of E. coli in Broths Containing Disrupted Cell Extracts and C. reinhardtii Cells

**Figure 4:**
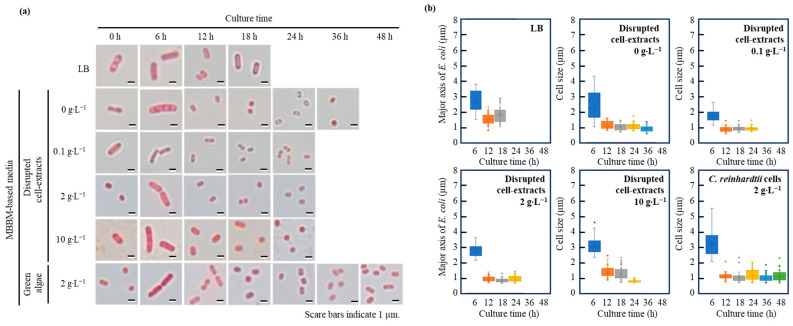
**Optical micrographs and cell size distributions of *E. coli* in each medium.** Morphological analyses for *E. coli* were performed in each broth. (**a**) Micrographs of *E. coli* were taken in the LB medium at 0 h, 6 h, 12 h and 18 h, in MBBM-based media containing disrupted cell extracts (0 g∙L^−1^; 0.1 g∙L^−1^; 2 g∙L^−1^;10 g∙L^−1^) at 0 h, 6 h, 12 h, 18 h and 24 h, and *C. reinhardtii* cells (2 g∙L^−1^) at 0 h, 6 h, 12 h, 18 h, 24 h, 36 h and 48 h. (**b**) Distributions of cells in each broth were analyzed over time (n = 30 (each time and broth)). The colors of blue, orange, gray, yellow, cyan and green meant 6 h, 12 h, 18 h, 24 h, 36 h and 48 h.

The cell sizes of *E. coli*, which were susceptible to nutrient starvation, were analyzed and evaluated in MBBM containing the disrupted cell extract or the *C. reinhardtii* cells (Figure 4). The major and minor axes of cells of *E. coli* cells were 2.6 ± 0.7 µm and 0.8 ± 0.1 µm at 6 h in the logarithmic growth phase in a nutrient-rich LB medium. Furthermore, the sizes of *E. coli* became slightly smaller, 1.8 ± 0.5 µm and 0.7 ± 0.1 µm as the major and minor axes during the stationary phase at 18 h, than the sizes at 6 h, especially showing that the sizes at 18 h were significantly different than those at 6 h (*p* *** < 0.001 (*p* = 1.8 × 10^−7^)). In the system of MBBM without the distributed cell extracts (0 g∙L^−1^) at 12 h, the major and minor axes of *E. coli* cells were 1.1 ± 0.2 µm and 0.7 ± 0.1 µm, and were not expanded larger during the culture time. In the system of MBBM with the distributed cell extracts (0.1 g∙L^−1^, 2 g∙L^−1^ and 10 g∙L^−1^) at 12 h, those sizes were 0.9 ± 0.2 µm and 0.5 ± 0.1 µm, 0.9 ± 0.2 µm and 0.6 ± 0.1 µm, and 1.3 ± 0.4 µm and 0.7 ± 0.1 µm, respectively. Although the major axes of *E. coli* cells in 0.1 g∙L^−1^ distributed cell extract-MBBM did not expand even by increasing the concentration of the distributed cell extract-MBBM to 2 g∙L^−1^ (*p* > 0.05 (*p* = 0.33)), those in 2 g∙L^−1^ distributed cell extract-MBBM significantly expanded by increasing the concentration of the distributed cell extract-MBBM to 10 g∙L^−1^ (*p* *** < 0.001 (*p* = 2.0 × 10^−7^)). Moreover, the major axes of *E. coli* cells in 10 g∙L^−1^ distributed cell extract-MBBM did not show a significant difference to those in the LB medium (*p* > 0.05 (*p* = 0.10)). Additionally, in the system of MBBM with *C. reinhardtii* (2 g∙L^−1^) at 12 h, the major and minor axes of *E. coli* were 1.2 ± 0.3 µm and 0.7 ± 0.1 µm. At 12 h, the major axes in *C. reinhardtii*-MBBM were significantly longer than those in 2 g∙L^−1^ distributed cell extract-MBBM (*p* *** < 0.001 (*p* = 3.8 × 10^−5^)) and shorter than those in 10 g∙L^−1^ distributed cell extract-MBBM (*p* ** < 0.01 (*p* = 6.8 × 10^−3^)). In both systems of the distributed cell extracts or the *C. reinhardtii* cells, the cell sizes reached the maximum at 6 h.

### 3.5. Transcriptomic Analysis for E. coli in C. reinhardtii-Containing MBBM

**Figure 5:**
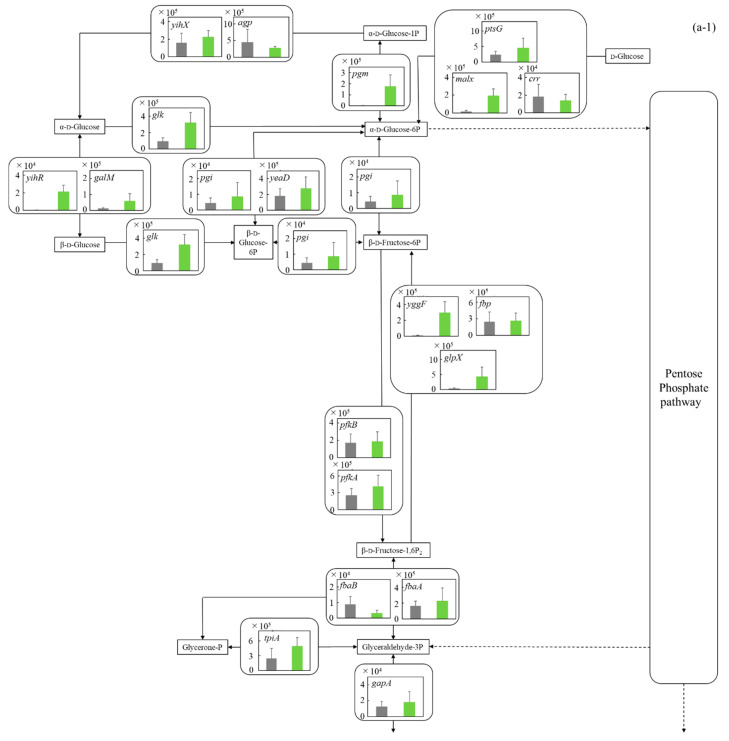

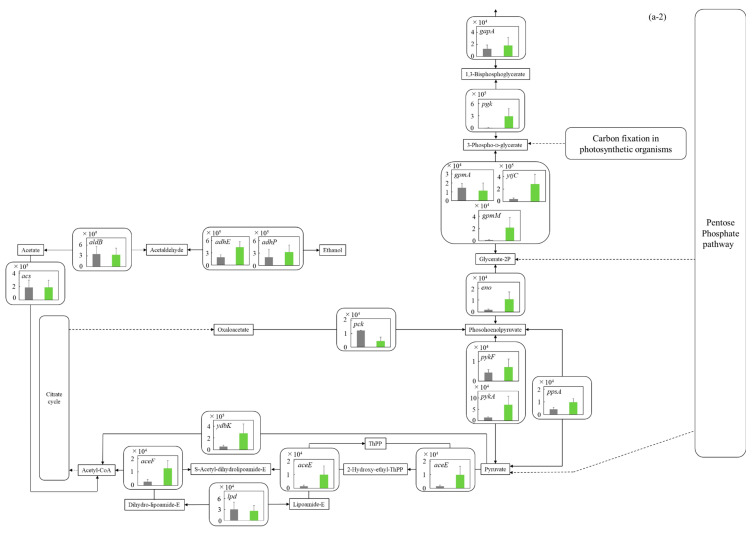

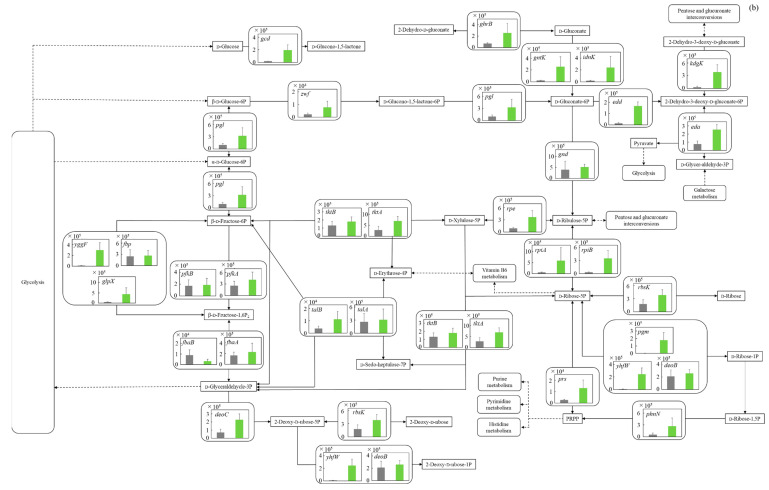

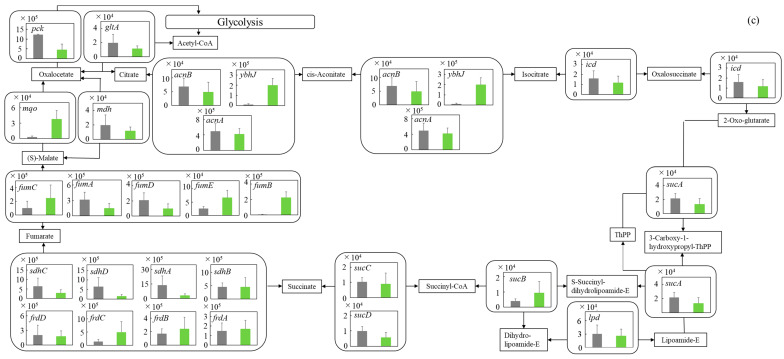

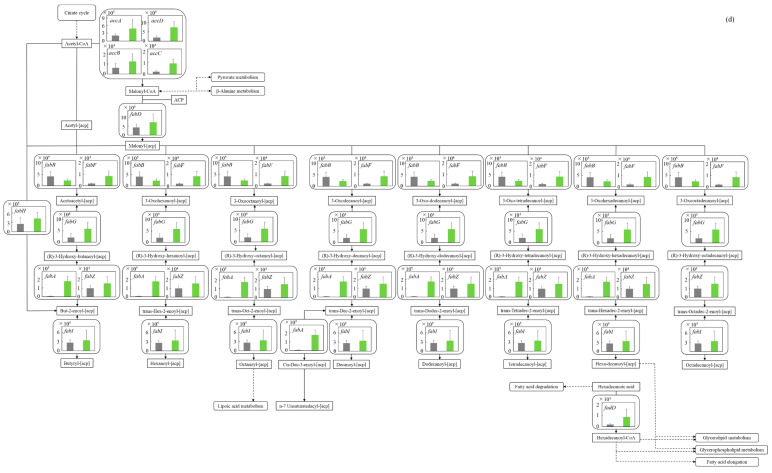

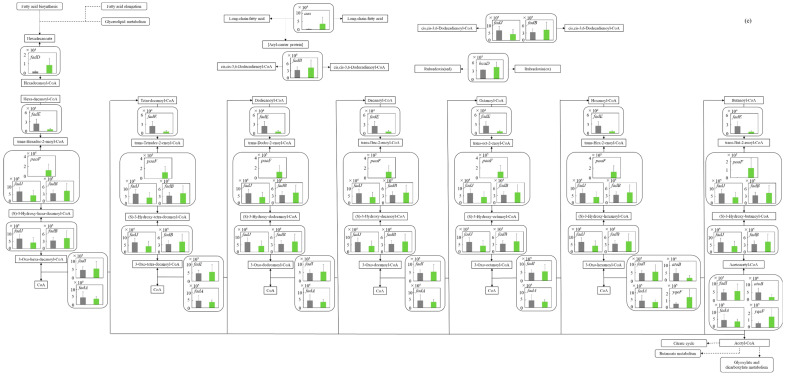

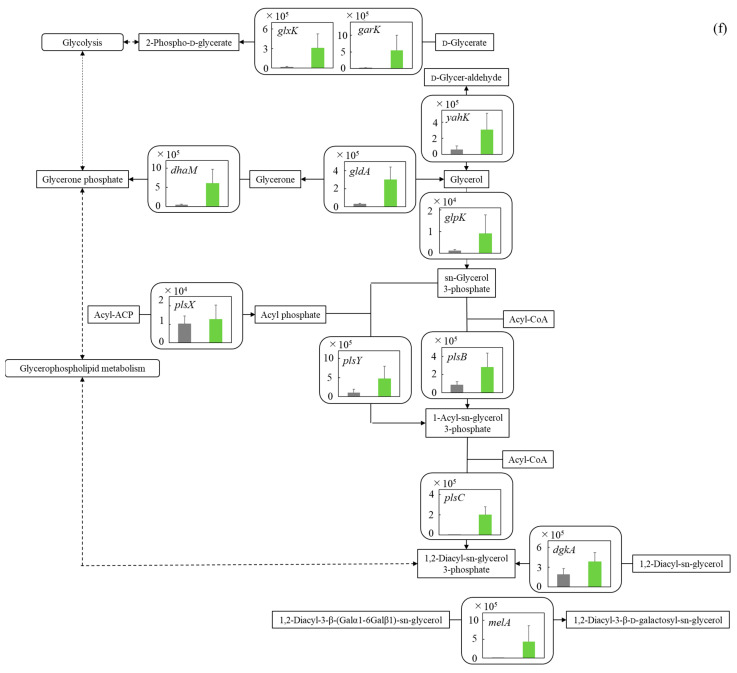
**Comparison of gene transcription levels of *E. coli* in MBBM containing *C. reinhardtii* cell and LB medium in glycolysis, pentose phosphate pathway, TCA cycle, fatty acid synthetic pathway, fatty acid degradation pathway, and glycerol lipid pathway. (a-1,a-2) in glycolysis.** Data are shown as relative mRNA transcription levels normalized by the level of *rrsA* as a housekeeping gene. Relative transcription levels in the LB medium and *C. reinhardtii*-MBBM at logistic phase are shown in black and green, respectively. Error bars indicate SD of 3~8 time replicate experiments. Gene abbreviations were shown as below: *glk*: *glucokinase*, *agp*: *glucose-1-phosphatase*, *yihX*: *glucose-1-phosphatase*, *pgm*: *phosphoglucomutase*, *ptsG*: *glucose PTS system EIICB or EIICBA component*, *malx*: *maltose/glucose PTS system EIICB component*, *crr*: *sugar PTS system EIIA component*, *galM*: *galactose-1-epimerase*, *yihR*: *aldose 1-epimerase*, *yeaD*: *glucose-6-phosphate 1-epimerase*, *pgi*: *glucose-6-phosphate isomerase*, *yggF*: *fructose-1,6-bisphosphatase II*, *glpX*: *fructose-1,6-bisphosphatase II*, *fbp*: *fructose-1,6-bisphosphatase I*, *pfkB*: *6-phosphofructokinase 2*, *pfkA*: *6-phosphofructokinase 1*, *fbaB*: *fructose-bisphosphate aldolase, class I*, *fbaA*: *fructose-bisphosphate aldolase*, *class II*, *tpiA*: *triose-phosphate isomerase*, *gapA*: *glyceraldehyde-3-phosphate dehydrogenase A*, *pgk*: *phosphoglycerate kinase*, *gpmA*: *2,3-bisphosphoglycerate-dependent phosphoglycerate mutase*, *ytjC*: *putative phosphatase*, *gpmM*: *2,3-bisphosphoglycerate-independent phosphoglycerate mutase*, *eno*: *enolase*, *pck*: *phosphoenolpyruvate carboxykinase (ATP)*, *pykF*: *pyruvate kinase I*, *pykA*: *pyruvate kinase II*, *ydbK*: *pyruvate-ferredoxin/flavodoxin oxidoreductase*, *ppsA*: *phosphoenolpyruvate synthetase*, *acs*: *acetyl-CoA synthetase (AMP-forming)*, *aceF*: *pyruvate dehydrogenase E2 component (dihydrolipoamide acetyltransferase)*, *aceE*: *pyruvate dehydrogenase E1 component*, *lpd*: *lipoamide dehydrogenase*, *aldB*: *aldehyde dehydrogenase B*, *adhE*: *acetaldehyde dehydrogenase/alcohol dehydrogenase*, *adhP*: *ethanol dehydrogenase/alcohol dehydrogenase*. **(b) in pentose phosphate pathway.** Data are shown as relative mRNA transcription levels normalized by the level of *rrsA* as a housekeeping gene. Relative transcription levels in the LB medium and *C. reinhardtii*-MBBM at logistic phase are shown in black and green, respectively. Error bars indicate SD of 3~8 time replicate experiments. Gene abbreviations were shown as below: *gcd*: *quinoprotein glucose dehydrogenase*, *ghrB*: *glyoxylate reductase*, *gntK*: *D-gluconate kinase, thermostable*, *idnK*: *D-gluconate kinase, thermosensitive*, *kdgK*: *2-dehydro-3-deoxygluconokinase*, *zwf*: *NADP(+)-dependent glucose-6-phosphate dehydrogenase*, *pgl*: *6-phosphogluconolactonase*, *edd*: *phosphogluconate dehydratase*, *eda*: *KHG/KDPG aldolase*, *gnd*: *6-phosphogluconate dehydrogenase*, *decarboxylating*, *tktB*: *transketolase 2*, *tktA*: *transketolase 1*, *rpe*: *ribulose-phosphate 3-epimerase*, *rpiA*: *ribose 5-phosphate isomerase A*, *rpiB*: *allose-6-phosphate isomerase/ribose-5-phosphate isomerase B*, *talB*: *transaldolase B*, *talA*: *transaldolase A*, *rbsK*: *ribokinase*, *yhfW*: *phosphopentomutase*, *deoB*: *phosphopentomutase*, *prs*: *ribose-phosphate pyrophosphokinase*, *phnN*: *ribose 1,5-bisphosphokinase*, *deoC*: *deoxyribose-phosphate aldolase*, *pgm*: *phosphoglucomutase*, *yggF*: *fructose-1,6-bisphosphatase II*, *glpX*: *fructose-1,6-bisphosphatase II*, *fbp*: *fructose-1,6-bisphosphatase I*, *pfkB*: *6-phosphofructokinase 2*, *pfkA*: *6-phosphofructokinase 1*, *fbaB*: *fructose-bisphosphate aldolase*, *class I*, *fbaA*: *fructose-bisphosphate aldolase, class II*. **(c) in TCA cycle.** Data are shown as relative mRNA transcription levels normalized by the level of *rrsA* as a housekeeping gene. Relative transcription levels in the LB medium and *C. reinhardtii*-MBBM at logistic phase are shown in black and green, respectively. Error bars indicate SD of 3~8 time replicate experiments. Gene abbreviations were shown as below: *mdh*: *malate dehydrogenase*, *mqo*: *quinone oxidoreductase*, *gltA*: *citrate synthase*, *acnB*: *aconitate hydratase 2/2-methylisocitrate dehydratase*, *ybhJ*: *putative hydratase YbhJ*, *acnA*: *aconitate hydratase 1*, *icd*: *isocitrate dehydrogenase*, *sucA*: *2-oxoglutarate dehydrogenase E1 component*, *sucB*: *dihydrolipoyltranssuccinylase*, *sucC*: *succinyl-CoA synthetase beta subunit*, *sucD*: *succinyl-CoA synthetase alpha subunit*, *sdhC*: *succinate:quinone oxidoreductase*, *membrane protein SdhC*, *sdhD*: *succinate:quinone oxidoreductase*, *membrane protein SdhD*, *sdhA*: *succinate:quinone oxidoreductase*, *FAD binding protein*, *sdhB*: *succinate:quinone oxidoreductase*, *iron-sulfur cluster binding protein*, *frdD*: *fumarate reductase membrane protein FrdD*, *frdC*: *fumarate reductase membrane protein FrdC*, *frdB*: *fumarate reductase iron-sulfur protein*, *frdA*: *fumarate reductase flavoprotein subunit*, *fumC*: *fumarate hydratase, class II*, *fumA*: *fumarate hydratase, class I, fumD*: *fumarate hydratase D*, *fume: fumarate hydratase E*, *fumB*: *fumarate hydratase, class I, pck*: *phosphoenolpyruvate carboxykinase (ATP)*, *lpd*: *lipoamide dehydrogenase*. **(d) in fatty acid synthetic pathway.** Data are shown as relative mRNA transcription levels normalized by the level of *rrsA* as a housekeeping gene. Relative transcription levels in the LB medium and *C. reinhardtii*-MBBM at logistic phase are shown in black and green, respectively. Error bars indicate SD of 3~8 time replicate experiments. Gene abbreviations were shown as below: *accA*: *acetyl-CoA carboxyltransferase subunit alpha*, *accD*: *acetyl-CoA carboxyltransferase subunit beta*, *accB*: *biotin carboxyl carrier protein*, *accC*: *biotin carboxylase*, *fabD*: *[acyl-carrier-protein] S-malonyltransferase*, *fabH*: *3-oxoacyl-[acyl-carrier-protein] synthase III*, *fabB*: *3-oxoacyl-[acyl-carrier-protein] synthase I*, *fabF*: *3-oxoacyl-[acyl-carrier-protein] synthase II*, *fabG*: *3-oxoacyl-[acyl-carrier protein] reductase*, *fabA*: *beta-hydroxyacyl-acyl carrier protein dehydratase/isomerase*, *fabZ*: *3-hydroxyacyl-[acyl-carrier-protein] dehydratase*, *fabI*: *enoyl-[acyl-carrier protein] reductase I*, *fadD*: *long-chain acyl-CoA synthetase*. **(e) in fatty acid degradation pathway.** Data are shown as relative mRNA transcription levels normalized by the level of *rrsA* as a housekeeping gene. Relative transcription levels in the LB medium and *C. reinhardtii*-MBBM at logistic phase are shown in black and green, respectively. Error bars indicate SD of 3~8 time replicate experiments. Gene abbreviations were shown as below: *fadE*: *acyl-CoA dehydrogenase*, *paaF*: *putative 2,3-dehydroadipyl-CoA hydratase*, *fadJ*: *3-hydroxyacyl-CoA dehydrogenase FadJ*, *fadB*: *3-hydroxyacyl-CoA dehydrogenase*, *fadI*: *3-ketoacyl-CoA thiolase FadI*, *fadA*: *3-ketoacyl-CoA thiolase*, *atoB*: *acetyl-CoA acetyltransferase*, *yqeF*: *putative acyltransferase*, *aas*: *fused 2-acylglycerophospho-ethanolamine acyltransferase/acyl-acyl carrier protein synthetase*, *hcaD*: *putative 3-phenylpropionate/cinnamate dioxygenase ferredoxin reductase subunit*. **(f) in glycerol lipid pathway.** Data are shown as relative mRNA transcription levels normalized by the level of *rrsA* as a housekeeping gene. Relative transcription levels in the LB medium and *C. reinhardtii*-MBBM at logistic phase are shown in black and green, respectively. Error bars indicate SD of 3~8 time replicate experiments. Gene abbreviations were shown as below: *glxK*: *glycerate 2-kinase 2*, *garK*: *glycerate 2-kinase 1*, *yahK*: *aldehyde reductase*, *NADPH-dependent*, *dhaM*: *dihydroxyacetone kinase subunit M*, *gldA*: *L-1,2-propanediol dehydrogenase/glycerol dehydrogenase*, *glpK*: *glycerol kinase*, *plsX*: *putative phosphate acyltransferase*, *plsY*: *putative glycerol-3-phosphate acyltransferase*, *plsB*: *glycerol-3-phosphate 1-O-acyltransferase*, *plsC*: *1-acylglycerol-3-phosphate O-acyltransferase PlsC*, *dgk*A: *diacylglycerol kinase (ATP)*, *melA*: *alpha-galactosidase*.

The gene transcription levels of *E. coli* in 2 g∙L^−1^ *C. reinhardtii*-MBBM were comprehensively analyzed compared to those cultured in the LB medium to evaluate the cellular responses of *E. coli* cultured with *C. reinhardtii* cells (Figure 5). As a first step, picking housekeeping genes up was attempted in order to evaluate the intracellular gene transcription levels using a relative quantitative method. According to the previous report, *16S ribosomal RNA* (*rrsA*) [24] is known to be constitutively expressed in cells and has actually been used as a normalizer (reference gene) for qPCR. Therefore, the transcription level was evaluated to know the performance as a reference gene on the qPCR in this study. As a result of qPCR, *rrsA* was stably amplified from a certain amount of cDNA, and *rrsA* was picked up as a housekeeping gene.

#### 3.5.1. Glycolysis and Ethanol Assimilating Pathway

The central metabolism from glucose to pyruvate is deeply involved in the metabolism (catabolism and assimilation) of carbon sources, and it is known that the metabolic system is activated during glucose catabolism [25] and glycerol assimilation [26]. The gene transcription levels in glycolysis of *E. coli* were comprehensively evaluated (Figure 5a-1,a-2). On the whole, in the metabolism from around glucose to pyruvate, the gene transcription levels in MBBM with *C. reinhardtii* were higher than those in the LB medium. In the cells in *C. reinhardtii*-MBBM compared to those in the LB medium, although the transcription level of *malX* (*p* ** < 0.01 (*p* = 0.002)) as d-glucose phosphorylational enzyme gene was 11.5 times higher with a significant difference, those of *ptsG* (*p* > 0.05 (*p* = 0.089)) and *crr* (*p* > 0.05 (*p* = 0.257)) showed no significant difference. Additionally, in the cells in *C. reinhardtii*-MBBM compared to those in the LB medium, the transcription level of *pgm* (*p* * < 0.05 (*p* = 0.015)) as the enzyme gene relating to the phosphoryl transfer of α-d-glucose 1P and α- d-glucose 6P was increased 103.7 times. Although the relevant gene transcript levels of *E. coli* in *C. reinhardtii*-MBBM did not show a large difference compared to those in the LB medium in the direction from α-d-glucose-6P to 1,3-bisphosphoglycerate, the transcription levels of *yggF* (*p* ** < 0.01 (*p* = 0.003)) and *glpX* (*p* ** < 0.01 (*p* = 0.007)) in *C. reinhardtii*-MBBM were 30.8 and 9.63 times higher than those in the LB medium in the direction from β-d-fructose-1,6P_2_ to β-d-fructose-6P. The transcription level of *pgk* (*p* ** < 0.01 (*p* = 0.003)) as the enzyme gene controlling the reactions of 1,3-bisphosphoglycerate and 3-phospho-d-glycerate was increased 43.1 times in the cells in *C. reinhardtii*-MBBM compared to those in the LB medium. In cells in *C. reinhardtii*-MBBM, the transcription levels of *aceE* (*p* ** < 0.01 (*p* = 0.006)) and *aceF* (*p* ** < 0.01 (*p* = 0.003)), as the enzyme genes in the metabolism from pyruvate to acetyl-CoA, were increased 6.40-fold and 4.46-fold compared to those in the LB medium.

#### 3.5.2. Pentose Phosphate Pathway

In the pentose phosphate pathway, which is an important metabolic pathway for nucleic acid synthesis deeply related to nearby glycolysis, the gene transcription levels were also evaluated (Figure 5b). In *C. reinhardtii*-MBBM compared to the LB medium, the transcription level of *gcd* (*p* ** < 0.01 (*p* = 0.003)) as the enzyme gene involved in the conversion reaction from d-glucose to d-glucono-1,5-lactone increased 10.1 times. Around d-gluconate-6P, all of the gene transcription levels in *C. reinhardtii*-MBBM were upregulated compared to those in the LB medium. Specifically, *zwf* (*p* > 0.05 (*p* = 0.068)) and *pgl* (*p* * < 0.05 (*p* = 0.030)) as the enzyme genes controlling the conversion of β-d-Glucose to d-gluconate-6P were upregulated 1.80 and 3.17 times; *gntK* (*p* * < 0.05 (*p* = 0.014)), *idnK* (*p* * < 0.05 (*p* = 0.035)), and *ghrB* (*p* * < 0.05 (*p* = 0.030)) as the enzyme genes regarding converting d-gluconate-6P to 2-dehydro-d-gluconate were 12.9, 15.9 and 3.68 times enhanced; *edd* (*p* *** < 0.001 (*p* = 0.0006)), *kdgK* (*p* ** < 0.01 (*p* = 0.007)) and *eda* (*p* *** < 0.001 (*p* = 0.0006)) as the enzyme genes relating to converting d-gluconate-6P to other pathways via 2-dehydro-d-gluconate were increased by 11.6, 12.6 and 3.28 times. In the pentose phosphate pathway, the transcription levels of enzyme genes relating d-xylulose-5P to d-ribose-1,5P via PRPP (5-phospho-d-ribosyl-1-diphosphate) were involved (*rpe* (*p* ** < 0.01 (*p* = 0.003)), 4.09 times; *rpiA* (*p* * < 0.05 (*p* = 0.038)) and *rpiB* (*p* ** < 0.01 (*p* = 0.006)), 10.2 times and 12.1 times; *prs* (*p* ** < 0.01 (*p* = 0.006)), 5.17 times; *phnN* (*p* * < 0.05 (*p* = 0.022)), 5.27 times) in *C. reinhardtii*-MBBM comparing to the LB medium. These genes were unique enzyme genes in each reaction pathway.

#### 3.5.3. TCA Cycle

The metabolic reactions in the TCA cycle are extremely important not only to prepare energy production but also to obtain amino acids such as arginine and alanine/aspartate/glutamate from 2-oxoglutarate and oxaloacetate in life. The gene transcription levels relating to tricarboxylic acid (TCA) cycle in *E. coli* were comprehensively evaluated (Figure 5c). In *C. reinhardtii*-MBBM, the gene transcription level of the enzyme gene *ybhJ* (*p* *** < 0.001 (*p* = 0.0009)) involved in the reaction between cis-aconitate and isocitrate was significantly upregulated 18.0 times rather than that in the LB medium. Furthermore, in *C. reinhardtii*-MBBM compared to in the LB medium, the transcription level of *fumB* (*p* ** < 0.01 (*p* = 0.008)), which is involved in the conversion reaction between fumarate and malate, was increased 40.1 times shown as significant difference; the gene transcription level of *mqo* (*p* ** < 0.01 (*p* = 0.003)) in *C. reinhardtii*-MBBM was significantly upregulated 13.1 times compared to that in the LB medium. However, all of the gene transcription levels did not show huge differences between the culture conditions in *C. reinhardtii*-MBBM and in the LB medium.

#### 3.5.4. Fatty Acid Synthetic Pathway

In alive organisms, the production of lipids has a very important meaning in the conservation of energy as an anabolic response. Therefore, the evaluation of the gene transcription levels related to the metabolic pathway has sufficient meaning to know the cellular responses to culture medium conversion. The gene transcription levels in fatty acid synthesis in *E. coli* were also evaluated comprehensively (Figure 5d). As the result shows, all gene transcription levels were increased in *C. reinhardtii*-MBBM compared to those in the LB medium. In the case of the culture in *C. reinhardtii*-MBBM towards LB medium, the gene transcription levels of *accA* (*p* > 0.05 (*p* = 0.052)), *accB* (*p* * < 0.05 (*p* = 0.022)), *accC* (*p* ** < 0.01 (*p* = 0.002)), and *accD* (*p* ** < 0.01 (*p* = 0.002)) as the genes of the enzymes converting acetyl-CoA to malonyl-CoA showed the trend to increase 2.34, 2.01, 4.36 and 4.05 times. The transcription levels of the enzyme genes of *fabF* (*p* ** < 0.01 (*p* = 0.006)), the elongation reaction of fatty acids from malonyl [acp], *fabG* (*p* * < 0.05 (*p* = 0.014)), NADPH-dependent reduction in β-keto acyl-acyl carrier protein, and *fabA* (*p* ** < 0.01 (*p* = 0.002)) and enoylation increased 4.58, 2.83 and 46.6 times. On the other hand, no significant difference was displayed in the *fabH* (*p* > 0.05 (*p* = 0.089)) transcription levels in both *C. reinhardtii*-MBBM and the LB medium.

#### 3.5.5. Fatty Acid Degradation Pathway

In alive organisms, the degradation of lipids has also a very important meaning in the production of energy as a catabolic response. As the cellular responses, whether the cells attempted to synthesize or degrade lipids was evaluated by analyzing the shift in the transcription levels of the genes relating to the lipid degradation compared to those involved in lipid synthesis. The gene transcription levels of fatty acid degradation in *E. coli* were comprehensively evaluated (Figure 5e). In both *C. reinhardtii*-MBBM and the LB medium, overall, the gene transcription levels in lipid degradation systems were not significantly different from those in lipid synthesis systems. Moreover, although the transcription level of *paaF* (*p* > 0.05 (*p* = 0.094)) of a gene of the enzyme involved in the intermediate process of lipid degradation increased 128 times, the transcription level of *fadE* (*p* * < 0.05 (*p* = 0.041)) of a gene of the unique enzyme involved in the first oxidation (generating an FADH_2_ molecule) in lipid degradation decreased 0.247 times. Furthermore, the transcription level of *fadD* (*p* > 0.05 (*p* = 0.101)) increased 4.65 times in *C. reinhardtii*-MBBM compared to the LB medium.

#### 3.5.6. Glycerolipid Pathway

Relating to the assimilating metabolic pathway, the gene transcription levels in the pathway converting fatty acid into lipids were also evaluated, comprehensively. The gene transcription levels of glycerolipid metabolism in *E. coli* were comprehensively evaluated (Figure 5f). In *C. reinhardtii*-MBBM compared to the LB medium, the transcription levels of *glxK* (*p* > 0.05 (*p* = 0.051)) and *garK* (*p* > 0.05 (*p* = 0.121)) genes, which are enzymes involved in the reaction from d-glycerate to enter the glycolysis, increased 23.4 and 26.0 times. The transcription levels of the genes *dhaM* (*p* ** < 0.01 (*p* = 0.003)), *plsB* (*p* ** < 0.01 (*p* = 0.007)), and *plsC* (*p* *** < 0.001 (*p* = 0.0005)), which are enzymes involved in oil and fat synthesis, in *C. reinhardtii*-MBBM were higher 13.1, higher 3.32 and 640 times than those in the LB medium.

## 4. Discussion

As shown in Figure 1a, the results strongly proved that *E. coli* could utilize the disrupted cell extracts as a nutrient source. In addition, doubling the exposure time of *E. coli* from 6 to 12 h tended to lessen its dependence on the concentration of disrupted cell extracts, indicating that increasing the disrupted cell extracts could improve the supply of nutrients for the cell growth of *E. coli* (Table 1). However, the alive cell densities did not increase after 12 h in MBBMs containing the disrupted cell extracts in this study case, meaning that *E. coli* exhausted the extracts up to 10 g∙L^−1^ concentration for 12 h. Additionally, the cell density reached a plateau at 12 h, and there seemed to be a correlation between the alive cell densities and the concentrations of disrupted cell extracts. To reveal the degree of the correlation, those values were plotted and the standard curve was analyzed (Figure 1b). The high correlation (*R*^2^ = 0.996) could indicate that the alive cell densities of *E. coli* were enhanced, depending on the increased concentrations of the disrupted cell extracts. Normally, when cells are provided with an excess amount of nutrients for growth, they cannot fully utilize the surrounding nutrients, resulting in the cells demonstrating a growth rate lower than the expected growth rate correlating to the excess nutrient concentration as its maximum growth rate. In this study, the viable cell density of *E. coli* did not decrease even at a concentration of the disrupted cell extracts of 10 g∙L^−1^ for 12 h. Therefore, the growth rate of *E. coli* did not reach the maximum concentration because the concentration of the cell extracts as nutrients was insufficient, and the growth rate of the supply of the extracts could be possibly further improved.

At 12 h, the alive cell density of *E. coli* in *C. reinhardtii*-MBBM was not significantly different from that in 10 g∙L^−1^ disrupted cell extract-MBBM (Figure 2a, Table 1, Supplemental Figure S1), meaning that those showed the equivalent growth pattern. Furthermore, from 6 h to 12 h, the specific growth rate and the doubling time of *E. coli* in *C. reinhardtii*-MBBM were also no significant difference to those in 10 g∙L^−1^ disrupted cell extract-MBBM, indicating the same growth pattern of *E. coli* with *C. reinhardtii*. Therefore, *E. coli* in *C. reinhardtii*-MBBM was supplied with nutrients from *C. reinhardtii* cells equivalent to 10 g∙L^−1^ disrupted cell extracts without growth inhibition in the log phase as the active growth phase. Compared to the *µ_max_* (approximately 1.2 ± 0.4 h^−1^) of *E. coli* such as MG1655 in the LB medium [27], the *µ_max_* of *E. coli* obtained the nutrition from *C. reinhardtii* cells in this study (1.04 ± 0.27 h^−1^) was typical value in the phase.

The alive cell density of *E. coli* in *C. reinhardtii*-MBBM significantly increased after 12 h, indicating that *C. reinhardtii* possibly continued to supply metabolites as the nutritional sources to *E. coli*; however, it was unclear whether alive or dead *C. reinhardtii* continued to supply metabolites. As a result of the analysis, there were no significant differences between the density and the survival ratio, and therefore there was no significant difference in the alive cell density in MBBM whether with or without *E. coli*, revealing that *C. reinhardtii* did not grow regardless of the presence or absence of *E. coli* not in the PBR system (Figure 2b). The above results indicated that *C. reinhardtii* did not die due to coexistence with *E. coli* but rather due to the culture environment which lacked light and aeration. In addition, the proliferation of *E. coli* remained in 2 g∙L^−1^ *C. reinhardtii*-MBBM, even after 12 h, while it stopped in 2 g∙L^−1^ disrupted cell extract-MBBM after 12 h (Figure 1a and Figure 2a; Table 1); therefore, the maximum cell density of *E. coli* in *C. reinhardtii*-MBBM was significantly higher than that in the disrupted cell extract-MBBM, indicating that *E. coli* could be continuously supplied with the nutrient sources directly from *C. reinhardtii* cells. The decrement in the cell survival ratio of *C. reinhardtii* was stopped to about 20% until 24 h and maintained after 24 h; however, the increment of alive cell density of *E. coli* kept until 36 h, strongly suggesting that *C. reinhardtii* not only provided the nutrient sources to *E. coli* via the cell rupture caused by death, but also supplied the nutrient source while being alive.

In 2 g∙L^−1^ disrupted cell extract MBBM, until 12 h, when the growth of *E. coli* almost stops, although the carbohydrate concentration showed few differences, the concentrations of protein, formic acid, acetic acid, and citric acid displayed a decrement (Figure 3). In fact, *E. coli* is known to assimilate amino acids [28], formic acid [29], acetic acid [30], and citric acid [31], and the results obtained here indicated the possibility that these nutrients were used for the growth of *E. coli*. Regarding the citric acid concentration after 18 h, its presence could no longer be confirmed until 24 h. The result may indicate that *E. coli* maintained its metabolism when using citric acid with few proliferations. Regarding lipids, no increase or decrease was observed while there was no reason for the lipid to actively increase in the culture medium, so it seems that lipid is not actively used by *E. coli*. In 2 g∙L^−1^ *C. reinhardtii*-MBBM, the protein was not detected at 0 h, but the concentration gradually increased up to 18 h. In 2 g∙L^−1^ disrupted cell extract MBBM, the protein consumption by *E. coli* was suggested; however, in 2 g∙L^−1^ *C. reinhardtii*-MBBM, the protein concentration was guaranteed with continuous protein releasing by *C. reinhardtii* beyond the consumption of *E. coli*. At 0 h, while carbohydrate was detected in 2 g∙L^−1^ disrupted cell extract MBBM, that could not be measured in 2 g∙L^−1^ *C. reinhardtii*-MBBM. The results simply indicated that *C. reinhardtii* cells contained carbohydrates; however, the cells could not release carbohydrates in the begining point of the culture. At 6 h, the carbohydrate concentration at 2 g∙L^−1^ *C. reinhardtii*-MBBM almost reached the concentration at 2 g∙L^−1^ of disrupted cell extract; after 6 h, those concentrations became stable. As a result, the amount of carbohydrate obtained up to 6 h was all that was obtained from *C. reinhardtii* cells, indicating that the carbohydrates could not have been consumed. On the other hand, interesting points were that formic acid and citric acid were detected in the medium even at 0 h as the starting point of culturing. These organic substances may be easily released to the outside from cells of *C. reinhardtii* by environmental changes such as altering the culture medium. Formic acid concentration did not shift over 12 h of cultivation; however, the formic acid was consumed during the growth of *E. coli* in 2 g∙L^−1^ disrupted cell extract-MBBM, and *E. coli* continued to proliferate in 2 g∙L^−1^ *C. reinhardtii*-MBBM after 12 h of culturing, meaning that the formic acid could be consumed by *E. coli* while approximately the same amount of formic acid continued to be supplied by *C. reinhardtii*. In the same way, the citric acid could not only be consumed by *E. coli* but also be continuously supplied by *C. reinhardtii* cells because the concentration was maintained in 2 g∙L^−1^ *C. reinhardtii*-MBBM under cultivation. In the case of acetic acid, the reason why the existence of acetic acid had not been detected from the beginning of cultivation may be because the acetic acid existed inside *C. reinhardtii* cells and it is difficult to access the outside. Furthermore, there was a possibility that the quantity could not be detected in the medium since acetic acid could also be consumed by *E. coli* even though acetic acid was released from *C. reinhardtii* cells into the culture medium. The concentration of lipids in the medium decreases slightly, meaning that lipid scould not be actively used by *E. coli*.

The sizes of the *E. coli* changes depend on the nutritional conditions and decrease with starvation [32]. The sizes of *E. coli* in the logarithmic growth phase and the stationary phase shown in the LB medium fall within the generally reported sizes of *E. coli* (maximum axis approximately 2.5~6.0 µm; minimum axis approximately 0.8~1.2 µm) [33]; these *E. coli* cells maintained their cell size for 18 h and could be used as a control for the comparison under a non-nutrient starvation condition in this study (Figure 4). On the other hand, the sizes of *E. coli* without the distributed cell extracts (0 g∙L^−1^) decreased by 12 h and did not increase thereafter, and could be used as a control for the comparison under the nutrient starvation conditions. In the systems supplemented with the distributed cell extracts, the sizes of *E. coli* cells were larger, as the concentration increased at 12 h when proliferations were about to stop, indicating that the nutritional conditions of *E. coli* cells were improved by increasing the concentrations of the distributed cell extracts. In a system of adjusted 10 g∙L^−1^ of distributed cell extracts, the major axes of *E. coli* cells at 12 h size did not display significant differences towards those in the LB medium, meaning that the nutritional status in the system under 10 g∙L^−1^ of distributed cell extracts was sufficient enough to not show the size difference. Although the cell sizes in 2 g∙L^−1^ *C. reinhardtii*-MBBM at 12 h were significantly inferior to those in the LB medium and in 10 g∙L^−1^ of distributed cell extracts MBBM at 12 h, the cell sizes in 2 g∙L^−1^ *C. reinhardtii*-MBBM were significantly prior to those in 2 g∙L^−1^ distributed cell extracts MBBM. Thus, the evaluation strongly suggested that *E. coli* continuously obtained nutrients from *C. reinhardtii*. The reason why the cell sizes reached the maximum after 6 h in each system of MBBM with the distributed cell extracts or *C. reinhardtii* cells, respectively, might be that the cells were still in a healthy state containing nutrients in the cells from the LB medium as the pre-culture.

To date, several reports attempted to reveal the cellular responses and how the microbial cells tried to control those metabolisms in detail with the comprehensive analysis of gene transcription levels [34]. In this study, the comprehensive gene transcription levels were also analyzed in order to evaluate the details of cellular responses in the culture environment and to infer metabolic control (Figure 5).

*C. reinhardtii*-MBBM should contain carbon sources of intermediate metabolites that *E. coli* can use as nutrients derived from *C. reinhardtii* cells with high possibility [35]; on the other hand, the LB medium generally consists of 1% tryptone, 0.5% yeast extract and 1% NaCl, and the yeast extract contains carbon sources such as inosinic acid and guanylic acid at 7–13 wt%, resulting in the LB medium showing 0.035~0.065 wt% carbohydrate sources [36]. Evaluating the metabolic system by analyzing the comprehensive gene transcription levels of *E. coli*, all those in *C. reinhardtii*-MBBM were higher than those in the LB medium (Figure 5a-1,a-2). These results could mean that *C. reinhardtii*-MBBM could contain more carbon sources relating to central metabolism than LB medium; therefore, *E. coli* possibly responded by trying to utilize the carbon sources derived from *C. reinhardtii* cells in central metabolism. The fact that the transcript level of *malX* as the enzyme gene controlling the d-glucose phosphorylation was significantly higher in *C. reinhardtii*-MBBM could also indicate that the system attempts to take in and utilize carbohydrate sources. Furthermore, *pgk*-disrupted strains of *E. coli* cannot grow on sugars or gluconeogenic substrates [37], and *pgk* was exhibited to be induced over 10-fold during the transition from exponential to stationary growth phase [37], meaning that *pgk* is an essential gene for sugar metabolism in *E. coli*. In this study, the transcription level of *pgk* was upregulated in *C. reinhardtii*-MBBM compared to that in the LB medium, so there was a possibility that *E. coli* showed a cellular response to utilize the carbohydrate source in *C. reinhardtii*-MBBM. *E. coli* accumulates glycogen within its cells to conserve energy, and activated *pgm* is an enzyme gene that produces α-d-glucose 1P from α-d-glucose 6P in order to supply the raw material ADP-glucose [38]. The transcription level of *pgm* was significantly improved in *C. reinhardtii*-MBBM compared to the LB medium in this study, indicating the possibility that *E. coli* is trying to store the carbohydrate sources in cells as an energy source. Furthermore, it is known that the metabolism of β-d-fructose-1,6P_2_ to β-d-fructose-6P is activated rather β-d-fructose-6P to β-d-fructose-1,6P_2_ when *E. coli* assimilates carbohydrate sources [39]. With the analysis in this study, while the gene transcription levels of *pfkA* and *pfkB* showed no increment in *C. reinhardtii*-MBBM compared to the LB medium, those of *yggF* and *glpX* were enhanced, reinforcing the possibility that *E. coli* was trying to store the carbon sources as an energy source. In the case that *E. coli* cannot use the carbon sources like glucose as a catabolite, there might be the possibility of running out of nitrogen sources supported by a report that glucose becomes one of the worst carbon sources for *E. coli* on poor nitrogen sources due to suboptimal levels of cAMP [40]. In the catabolic reactions of *E. coli*, the metabolic reactions that transfer carbon sources from glycolysis to the TCA cycle are important regarding energy production [41] and raw material production of useful substances such as amino acids [42]. In *C. reinhardtii*-MBBM compared to the LB medium, the transcription levels of *aceE* and *aceF* were increased, showing the possibility that cellular response promotes the production of energy and substances derived from carbohydrate sources from glycolysis. Assimilation of ethanol requires that ethanol be incorporated into acetyl CoA via acetate. According to the previous literature reports, *E. coli* can assimilate ethanol as a carbon source [43], and the *E. coli* strain in this study might also attempt to assimilate ethanol in *C. reinhardtii*-MBBM. However, Cao et al. found that BL21(DE3), DH5α, Top10, and JM109 derived all from *E. coli* K-12 were unable to assimilate ethanol, and *E. coli* strain can finally assimilate ethanol with overexpression of *E. coli adhE* and *E. coli aldA* by genetic engineering [12].

Metabolic pathways surrounding ribonucleotide synthesis in *C. reinhardtii*-MBBM could be activated rather than those in the LB medium due to the increased transcription levels of unique enzyme genes (*rpe*; *rpiA* and *rpiB*; *prs*; *phnN*) (Figure 5b). In particular, NAD biosynthesis in *E. coli* usually proceeds with consumption of PRPP [44] so the enhancement of PRPP pathway is possibly advantageous for regulating the intracellular redox balance. Lyngstadaas et al. reported that the *rpe*-disrupted strain of *E. coli* was unable to use single pentose, and that rpe could play a central role in pentose utilization [45], and the increased gene transcription level of that in *C. reinhardtii*-MBBM might indicate the assimilation of pentose from *C. reinhardtii*. Moreover, although *rpiA* and *rpiB* have different sequences, they were shown to have the same activities in the cells [46]. Therefore, the similar increase in the transcription levels of both genes in this study could mean that *E. coli* attempted to use pentose-like substances derived from *C. reinhardtii*. According to what Hove-Jensen et al. reported, *phnN* has activity in phosphonate degradation and NAD biosynthesis pathways in *E. coli* [44]. In this study, the gene transcription levels surrounding *phnN* were upregulated in *C. reinhardtii*-MBBM compared to the LB medium, and the facts could indicate the activation in a metabolic pathway surrounding ribonucleotides as metabolites. In addition, the gene transcription levels of enzyme-promoting carbon source leakage from the pentose phosphate pathway to other pathways including d-glucono-1,5-lactone were increased in *C. reinhardtii*-MBBM compared to the LB medium. Overall, as shown in Figure 5a-1,a-2, the increments of the gene transcription levels of enzymes relating to saccharide metabolism in the pentose phosphate pathway were also revealed, suggesting that *E. coli* co-cultured with *C. reinhardtii* might have activated sugar metabolism.

In both *C. reinhardtii*-MBBM and the LB medium, although the gene transcription levels of *ybhJ* and *fumB* were increased, they were not unique enzyme genes because of existing complement genes that could not specifically control the reaction (Figure 5c). In the same way, the gene transcription level of *mqo* as a non-unique enzyme gene was enhanced, implying that it was difficult for only mqo specifically to control the related reaction by supporting the complement enzyme mdh. Therefore, overall, the gene transcription levels in the TCA cycle showed fewer differences than those in glycolysis. However, the previous study reported that mqo activity was the highest in exponential growth and decreased sensitivity after the onset of the stationary phase [47], indicating that the increased *mqo* transcription level in our study could mean an active trial to proliferate the cells. As described above, by using *C. reinhardtii*-MBBM as opposed to the LB medium, major changes caused by the unique enzymes controlling each reaction did not appear in the TCA cycle. Therefore, there was the possibility that *E. coli* was not trying to cause major changes in metabolic activity regarding energy and amino acid production as the cellular response.

What was noteworthy was that the overall gene transcription levels in *C. reinhardtii*-MBBM were increased compared to those in the LB medium (Figure 5d). Especially, the transcription levels of enzymes involved in the reactions from acetyl-CoA to malonyl-CoA (*accA*, *accB*, *accC*, and *accD*) and those involved in fatty acid elongation (*fabF*, *fabG*, and *fabA*) showed the trend to enhance, suggested that lipid production could be promoted. Furthermore, Yao et al. reported that *fabH* disruption in *E. coli* promoted dwarfing of cell size [30], and few differences in the *fabH* transcription levels between *C. reinhardtii*-MBBM and LB medium without significance in this study could mean that the cell sizes of *E. coli* were not significantly reduced even in *C. reinhardtii*-MBBM.

Although overall the transcription levels in the lipid degradation pathway in *C. reinhardtii*-MBBM showed fewer differences than those in the LB medium and in the lipid synthesis pathway (Figure 5e), *fadE* as a unique gene of an enzyme relating to the first oxidation with the generation of FADH_2_ in lipid degradation pathway significantly decreased. As the results shown in Figure 5d,e, there was a possibility that *E. coli* in *C. reinhardtii*-MBBM could respond to synthesizing lipids as the carbon assimilation as a priority rather than degrading them. In addition, the experimental strain *E. coli* LS5218, which was selected as a strain that metabolizes octanoate, actively metabolizes the reaction with fadD. fadD, connecting substrates such as octanoate and hexadecanoate to CoA, is a very important enzyme, and the Δ*fadD* strain is lethal [48]. The transcription level of *fadD* in *C. reinhardtii*-MBBM tended to be higher than that in the LB medium, and the culture condition using *C. reinhardtii* as a nutrient source could be an effective medium condition for the metabolism relating to lipids.

In *C. reinhardtii*-MBBM, the transcription levels of genes related to lipid synthesis were more dominant than those of lipid degradation. Both *glxK* and *garK* catalyze the phosphorylation of d-glycerate [49], and their activation promotes the production of 2-phospho-d-glycerate, which is a necessary substrate for lipid synthesis (Figure 5f). In this study, the increased transcription levels of *glxK* and *garK* in *C. reinhardtii*-MBBM could also be important in terms of increasing the lipid substrate. In addition, the transcription levels of *dhaM*, *plsB*, and *plsC* increased in *C. reinhardtii*-MBBM compared to the LB medium, suggesting that glycerolipid metabolism could be activated relating to the lipid synthesis as a cellular response. This could be because dhaM is a phosphotransferase system-like protein and component of dihydroxy acetone kinase [50], and plsB is the membrane-bound enzyme that catalyzes substrates in the first step of phospholipid synthesis, which synthesizes lysophosphatidic acid (LPA) from long-chain ACP and sn-glycerol-3 phosphate (G3P) [51]. Therefore, the enhancement of those gene transcription levels showed the possibility that *E. coli* prepared the lipid synthesis. Additionally, plsC catalyzes the second step in phospholipid biosynthesis, and is thought to function in close proximity to the first step catalyzed by sn-glycerol-3-phosphate acyltransferase [52], strongly suggesting that *plcC* took over the activation of *dhaM* and *PlsB* to promotes cellular lipid synthesis as a cellular response. From the above, *E. coli* could perform the cellular responses to reinforce the metabolic system that promotes lipid production in *C. reinhardtii*-MBBM rather than the LB medium.

## 5. Conclusions

This study evaluated whether the heterotrophic microorganism *E. coli*, which is beneficial for material production, could use the metabolites as the nutrients from autotrophic microorganism *C. reinhardtii*. The growth of *E. coli* in an autotrophic medium containing *C. reinhardtii* cells was evaluated, resulting in the specific growth rate being similar to that in the LB medium. The results proved that *E. coli* could efficiently use metabolites derived from *C. reinhardtii*. In addition, the comprehensive gene transcription levels of *E. coli* in an autotrophic medium supplemented with *C. reinhardtii* were also assessed by transcriptome analysis. As the results showed, the gene transcription levels related to glycolysis, PRPP and lipid synthesis were upregulated; those in the TCA cycle had no specific upregulation in the TCA cycle, but those in the process of lipid degradation were decreased. In particular, the increase in the transcription level of *pgm* in glycolysis and the decrease in that of *fadE* in lipid degradation indicated the carbon source assimilation as a cellular response of *E. coli* in cultivation with *C. reinhardtii* since these unique genes could regulate the metabolic direction. This was the first report to clarify the cellular response of *E. coli* supplied with nutrients from living *C. reinhardtii* from the point of view of cell proliferation, morphology and transcriptomics.

## Figures and Tables

**Table 1:** **Growth properties of *E. coli* in each medium**.

Added Resource		Maximum Alive Cell Density		Growth Rate			
		Time (h)	Alive Cell Density (×10^8^ cells∙mL^−1^)	Time Range (h)	Specific Growth Rate (*µ*_max_, h^−1^)	Doubling Time (h)	Corresponding Figure
Disrupted cell extracts	0.1 g∙L^−1^	12	0.03 ± 0.02	6~12	0.27 ± 0.10	2.75 ± 0.84	Figure 1a
				12~24	Growth not shown	Not calculated	
	1 g∙L^−1^	24	0.64 ± 0.59	6~12	0.45 ± 0.34	2.78 ± 2.71	Figure 1a
				12~24	0.03 ± 0.02	38.85 ± 32.51	
	2 g∙L^−1^	12	1.15 ± 0.17	6~12	0.63 ± 0.29	1.26 ± 0.55	Figure 1a
				12~24	Growth not shown	Not calculated	
	4 g∙L^−1^	24	1.80 ± 0.23	6~12	0.50 ± 0.11	1.44 ± 0.35	Figure 1a
				12~24	0.06 ± 0.05	11.76 ± 10.61	
	10 g∙L^−1^	12	4.59 ± 0.48	6~12	0.82 ± 0.27	0.90 ± 0.25	Figure 1a
				12~24	Growth not shown	Not calculated	
Green algae	2 g∙L^−1^	36	34.48 ± 2.44	6~12	1.04 ± 0.27	0.71 ± 0.22	Figure 2a
				12~24	0.12 ± 0.01	5.62 ± 0.61	

## Data Availability

Data are contained within the article.

## Supplementary Materials

The following supporting information can be downloaded at: https://www.mdpi.com/article/10.3390/microorganisms12030452/s1, Figure S1 Complementation of growth of *E. coli* in *C. reinhardtii*-MBBM with those in disrupted cell-extract-MBBM; Table S1 Primer pairs used on qPCR.
