# Supplementary material for: Evaluation of Cellular Responses of Heterotrophic Escherichia coli Cultured with Autotrophic Chlamydomonas reinhardtii as a Nutrient Source by Analyses Based on Microbiology and Transcriptome
Source: Microorganisms. 2024 Feb 23;12(3):452. doi: 10.3390/microorganisms12030452 (PMC10972114; doi:10.3390/microorganisms12030452)
Supplement: Supplementary file 1 [file microorganisms-12-00452-s001.zip › microorganisms-2862722-supplementary.pdf]

Supplemental Data

Supplemental Figure S1

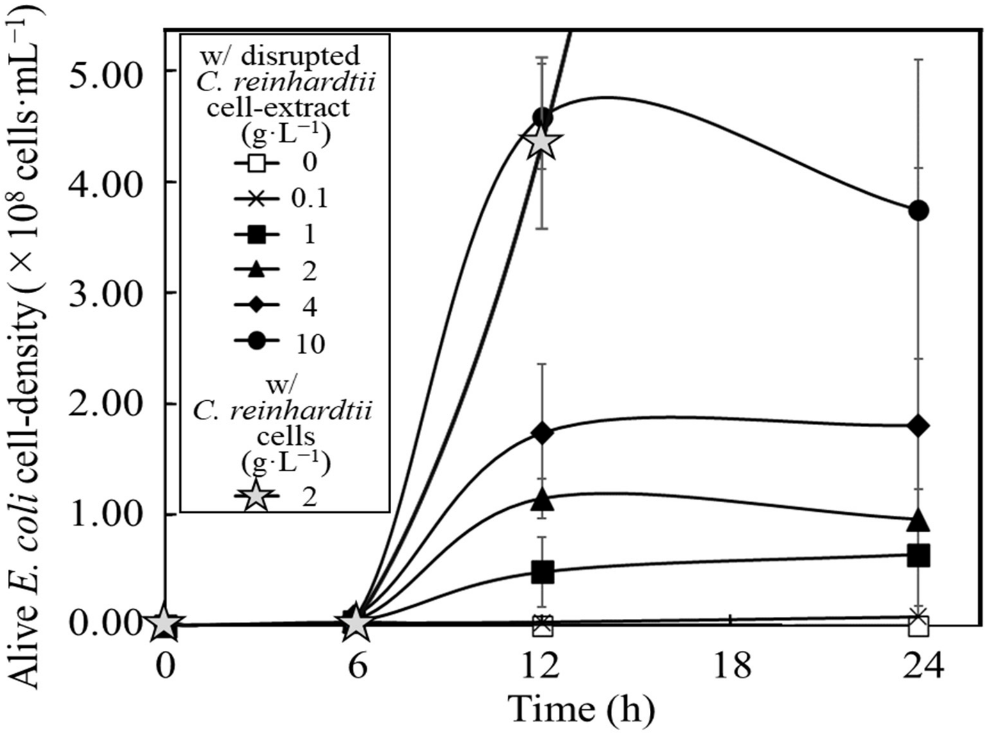

Supplemental Figure S1 Complementation of growth of *E. coli* in *C. reinhardtii*-MBBM with those in disrupted cell-extract-MBBM.
Those *E. coli* growth was compared using data depending on Figure 1 (a) and Figure 2 (a).
Growth activities of *E. coli* were shown in MBBM containing 0 (□), 0.1 (×), 1 (■), 2 (▲), 4 (◆) and 10 $g{\cdot}L^{-1}$ (●) of disrupted cell-extracts from *C. reinhardtii*, and in MBBM containing 2 $g{\cdot}L^{-1}$ (☆) of *C. reinhardtii* cells. Error bars indicate the SD of three replicate experiments (n = 3).

Supplemental Table S1. Primer pairs used on qPCR

Category	Enzyme name [EC number]	Abbreviation	Forward/ Reverse	Sequence alignment
Glycolysis	glucokinase [EC:2.7.1.2]	glk	F	agcggtcattcgcgtttatc
			R	gtcaccggtaattgggcaag
	glucose-1-phosphatase [EC:3.1.3.10]	agp	F	atctgaccagcagtggaagg
			R	ataactgaccagcggtttcg
	glucose-1-phosphatase [EC:3.1.3.10]	yihX	F	gtttttcacccagcgatacg
			R	aaatagtccgggatggtggt
	phosphoglucomutase [EC:5.4.2.2]	pgm	F	gcttctattggcggtctgaa
			R	ccgcttctttctcaatctgc
	glucose PTS system EIICB or EIICBA component [EC:2.7.1.199]	ptsG	F	tctttactggcgttgtgctg
			R	aaatgccaaacgcaactacc

maltose/glucose PTS system EIICB component [EC:2.7.1.199; 2.7.1.208]	malx	F<br>R	attacccgtctgcgtttgtc<br>actgaacttgtggcccgata
sugar PTS system EIIA component [EC:2.7.1.-]	crr	F<br>R	agacggcaccattggtaaaa<br>cgataccgaagtggacgaac
galactose-1-epimerase aldose 1-epimerase [EC:5.1.3.3]	galM	F<br>R	gccctgagttcagatgatgg<br>ggcgcgataagtaatggaga
putative sulfoquinovose mutarotase YihR aldose 1-epimerase [EC:5.1.3.3]	yihR	F<br>R	acggctatccgtttatgctg<br>agtgccgatattctggcttg
putative aldose 1-epimerase YeaD glucose-6-phosphate 1-epimerase [EC:5.1.3.15]	yeaD	F<br>R	tcgaacaaatctcccctgtc<br>gaggcttttacctggggatg
glucose-6-phosphate isomerase [EC:5.3.1.9]	pgi	F<br>R	caataccccgattttggttg<br>tcaccggaaataatcgcttc
fructose 1,6-bisphosphatase YggF fructose-1,6-bisphosphatase II [EC:3.1.3.11]	yggF	F<br>R	tatcggcgaaggagaaattg<br>gtgccttcaatgggatcaac
fructose-1,6-bisphosphatase 2 fructose-1,6-bisphosphatase II [EC:3.1.3.11]	glpX	F<br>R	tcgtcattggtgaaggtgaa<br>gtgccttcaatcggatcaac
fructose-1,6-bisphosphatase 1 fructose-1,6-bisphosphatase I [EC:3.1.3.11]	fbp	F<br>R	tctggatatcatcccggaaa<br>agcgttcgacatcttcaacc
6-phosphofructokinase 2 [EC:2.7.1.11]	pfkB	F<br>R	aactcagtgcgctggtgaat<br>tgccgctattaacgatttcc
6-phosphofructokinase 1 [EC:2.7.1.11]	pfkA	F<br>R	aattcagccgtgaagacctg<br>tttcacgaccggtttctttc
fructose-bisphosphate aldolase, class I [EC:4.1.2.13]	fbaB	F<br>R	ttgctgctaacccgctctac<br>cgtaagttgacgccacacag
fructose-bisphosphate aldolase, class II [EC:4.1.2.13]	fbaA	F<br>R	aatcggcatgactctggaaa<br>gtacagtgcagaagcgtcca
triose-phosphate isomerase triosephosphate isomerase (TIM) [EC:5.3.1.1]	tpiA	F<br>R	tgacgctaacatcgctgaac<br>atatccggctgagcaaacag
glyceraldehyde-3-phosphate dehydrogenase A glyceraldehyde 3-phosphate	gapA	F<br>R	agttgacctgaccgttcgtc<br>acgtcatcttcggtgtagcc

dehydrogenase (phosphorylating) [EC:1.2.1.12]			
phosphoglycerate kinase [EC:2.7.2.3]	pgk	F	acgaagctgacctggttgac
		R	ttgctacgcgaacatcagac
2,3-bisphosphoglycerate-dependent phosphoglycerate mutase [EC:5.4.2.11]	gpmA	F	aaaagtatggcgacgagcag
		R	gtcagcggcagttctttctc
putative phosphatase 2,3-bisphosphoglycerate-dependent phosphoglycerate mutase [EC:5.4.2.11]	ytjC	F	cggctgtgacatcatctttg
		R	cgacgccagttctcttcttc
2,3-bisphosphoglycerate-independent phosphoglycerate mutase [EC:5.4.2.12]	gpmM	F	ttgggatcgcgtagaaaaag
		R	ataagcagcctgcaaaccag
enolase [EC:4.2.1.11]	eno	F	gcgaaactgaagacgctacc
		R	acggtcagaacggctcatag
phosphoenolpyruvate carboxykinase (ATP) [EC:4.1.1.49]	pck	F	caaacgtctgttcgttgtcg
		R	aacctgccagttcttcatcg
pyruvate kinase I [EC:2.7.1.40]	pykF	F	caccgtactggttgacgatg
		R	ggcaggttcacacctttgtt
pyruvate kinase II [EC:2.7.1.40]	pykA	F	acatcatcctcgcctctgac
		R	tgattaccgctcggtttagc
pyruvate-ferredoxin/flavodoxin oxidoreductase [EC:1.2.7.1; 1.2.7.-]	ydbK	F	gtggtacgcacagggctatt
		R	caaaatcagcctgggaaatg
phosphoenolpyruvate synthetase pyruvate, water dikinase [EC:2.7.9.2]	ppsA	F	aacggatgaaagacggtgag
		R	attccagcaactccgcatag
acetyl-CoA synthetase (AMP-forming) [EC:6.2.1.1]	acs	F	ggcgcattattgattccaac
		R	gtgacgttcgggtttttcag
pyruvate dehydrogenase E2 component (dihydrolipoamide acetyltransferase) [EC:2.3.1.12]	aceF	F	tgctgaaaacgacgcttatg
		R	cgcaggatacgacctttacg
pyruvate dehydrogenase E1 component [EC:1.2.4.1]	aceE	F	actgggtactgatggcttcg
		R	atcgatttcgccacgtttag
lipoamide dehydrogenase dihydrolipoamide dehydrogenase [EC:1.8.1.4]	lpd	F	tcaccaagcgtatcagcaag
		R	tgccttccatcgtcacataa
aldehyde dehydrogenase B [EC:1.2.1.-]	aldB	F	taccccgctttctgtactgc
		R	ttcgaggtcgccagatattc
	adhE	F	cctgactctgggttgtggtt

acetaldehyde dehydrogenase / alcohol dehydrogenase [EC:1.2.1.10; 1.1.1.1]		R	ctcgcttagcaacggttttc
ethanol dehydrogenase/alcohol dehydrogenase [EC:1.1.1.1]	adhP	F<br>R	ggatatcccacgtcttgtgc<br>ttcggcaccactttaccttc
malate dehydrogenase [EC:1.1.1.37]	mdh	F<br>R	ttttaccgagcaggaagtgg<br>tgcacgaaccagagacagac
quinone oxidoreductase<br>malate dehydrogenase (quinone) [EC:1.1.5.4]	mqo	F<br>R	agtgctggggttgaactacg<br>tccgccacttctttttgaac
citrate synthase [EC:2.3.3.1]	gltA	F<br>R	atgattctttccgcctgatg<br>cccagctctttcagcacttc
aconitate hydratase 2 / 2-methylisocitrate dehydratase [EC:4.2.1.3 4.2.1.99]	acnB	F<br>R	aagtgcgtaaccacgaaacc<br>cagcacgcacttcatcaatc
putative hydratase YbhJ<br>aconitate hydratase [EC:4.2.1.3]	ybhJ	F<br>R	aaaatggtcgcctgaaagtg<br>gtaaactgccagcgagaagg
aconitate hydratase 1 [EC:4.2.1.3]	acnA	F<br>R	tatctcggcaaagcagtgtg<br>tgcgagtcagtaccaacgag
isocitrate dehydrogenase [EC:1.1.1.42]	icd	F<br>R	cccgtggctgaaagttaaaa<br>aggatctgttgcaggaatgc
2-oxoglutarate dehydrogenase E1 component [EC:1.2.4.2]	sucA	F<br>R	cttacctctctggcgcaaac<br>accaggtaactgctggaacg
dihydrolipoyltranssuccinylase<br>2-oxoglutarate dehydrogenase E2 component (dihydrolipoamide succinyltransferase) [EC:2.3.1.61]	sucB	F<br>R	tggtcgtctgactcgtgaag<br>gtcatcgggacacgtttttc
succinyl-CoA synthetase beta subunit [EC:6.2.1.5]	sucC	F<br>R	ggtccgtgggtagtgaaatg<br>gcacggatgtcttctttgct
succinyl-CoA synthetase alpha subunit [EC:6.2.1.5]	sucD	F<br>R	tgctgaccgtgaaagtgaag<br>cggtttgtgaatgtgaccag
succinate:quinone oxidoreductase, membrane protein SdhC<br>succinate dehydrogenase / fumarate reductase, cytochrome b subunit [EC: 1.3.5.1]	sdhC	F<br>R	cggtgtgatcacctttgttg<br>aagcttgctcgaaaccttca
succinate:quinone oxidoreductase, membrane protein SdhD<br>succinate dehydrogenase / fumarate	sdhD	F<br>R	cgtcctgacgctctacatca<br>atagaaaacagcgccagcag

	reductase, membrane anchor subunit [EC:1.3.5.1]			
	succinate:quinone oxidoreductase, FAD binding protein<br>succinate dehydrogenase / fumarate reductase, flavoprotein subunit [EC:1.3.5.1; 1.3.5.4]	sdhA	F	ggtcttccgtgaaggtgatg
			R	tgttgaactcgctggaagtg
	succinate:quinone oxidoreductase, iron-sulfur cluster binding protein<br>succinate dehydrogenase / fumarate reductase, iron-sulfur subunit [EC:1.3.5.1; 1.3.5.4]	sdhB	F	aaaactcgacgggctgtatg
			R	acgatatgccgctaacaagc
	fumarate reductase membrane protein FrdD<br>fumarate reductase subunit D	frdD	F	cttcattggtcgcgtattcc
			R	agcagccagaccgtagaaaa
	fumarate reductase membrane protein FrdC<br>fumarate reductase subunit C	frdC	F	gtttgaactggcaccgaaag
			R	gggcaacaaacaggattacg
	fumarate reductase iron-sulfur protein<br>fumarate reductase iron-sulfur subunit [EC:1.3.5.4]	frdB	F	gtatggcgatttgtggttcc
			R	acgcttcaaccttcataccg
	fumarate reductase flavoprotein subunit [EC:1.3.5.4]	frdA	F	cgaaatgacccaactggaac
			R	tatggaagccggtcttatcg
	fumarase C<br>fumarate hydratase, class II [EC:4.2.1.2]	fumC	F	cattacctgtgcaccgtttg
			R	ggacatcattggcgattttc
	fumarase A<br>fumarate hydratase, class I [EC:4.2.1.2]	fumA	F	ggggatctggatcgaaaaac
			R	aaacgggatactgcgacaac
	fumarase D<br>fumarate hydratase D [EC:4.2.1.2]	fumD	F	tgggaaatcggacaaaagag
			R	cgcctgcgataacaatatgc
	fumarase E<br>fumarate hydratase E [EC:4.2.1.2]	fumE	F	tgtgcttgagcaactggatg
			R	acggcttcactgcatattcc
	fumarase B<br>fumarate hydratase, class I [EC:4.2.1.2]	fumB	F	ccggtatccactcgtttgtc
			R	gaagttctttaccggcgtca
Pentose phosphate pathway	quinoprotein glucose dehydrogenase [EC:1.1.5.2]	gcd	F	actgtggcagggtcgtttac
			R	tagtcgcccatcttcgtacc
	glyoxylate reductase<br>glyoxylate/hydroxypyruvate/2-ketogluconate reductase [EC:1.1.1.79; 1.1.1.81; 1.1.1.215]	ghrB	F	atttcgtttgcctgatcctg
			R	cagtgcattttcgtcaacca
	D-gluconate kinase, thermostable gluconokinase [EC:2.7.1.12]	gntK	F	ttcttgatggcgatttcctc
			R	tagtgcgctgcatagcaaac

D-gluconate kinase, thermosensitive gluconokinase [EC:2.7.1.12]	idnK	F<br>R	ctggctggaacgcttaaatg<br>cgccatctaaccagaggaaa
2-dehydro-3-deoxygluconokinase [EC:2.7.1.45]	kdgK	F<br>R	tactactggcggaacgaagc<br>taatcgaaattcgccagctc
NADP(+)-dependent glucose-6-phosphate dehydrogenase glucose-6-phosphate 1-dehydrogenase [EC:1.1.1.49; 1.1.1.363]	zwf	F<br>R	cgtcgtaaattgctgccttc<br>tatgccgctttatcccagtc
6-phosphogluconolactonase [EC:3.1.1.31]	pgl	F<br>R	atccgcacggtaatatcgaa<br>cgtataaatggcgaccatcc
phosphogluconate dehydratase [EC:4.2.1.12]	edd	F<br>R	cgattgtgtcgttgttgtcc<br>ggagagtcgtccatcggtaa
KHG/KDPG aldolase 2-dehydro-3-deoxyphosphogluconate aldolase / (4S)-4-hydroxy-2-oxoglutarate aldolase [EC:4.1.2.14; 4.1.3.42]	eda	F<br>R	agaagtgcctgaagcgattg<br>ccttcggtagcagctttcag
6-phosphogluconate dehydrogenase, decarboxylating 6-phosphogluconate dehydrogenase [EC:1.1.1.44 1.1.1.343]	gnd	F<br>R	agatcggcgtagtcggtatg<br>tttcggcaatcacttcttcc
transketolase 2 transketolase [EC:2.2.1.1]	tktB	F<br>R	tacgccggatcaggttaaag<br>gctaatttctctgccgcttg
transketolase 1 transketolase [EC:2.2.1.1]	tktA	F<br>R	acggctccatgctgatctac<br>gcagctgacggaagttttc
ribulose-phosphate 3-epimerase [EC:5.1.3.1]	rpe	F<br>R	aagtacgtcgccgtatcgac<br>gtctggctggtcgaagattg
ribose 5-phosphate isomerase A [EC:5.3.1.6]	rpiA	F<br>R	ctgtattgcagacgcttcca<br>ctacgtgccatcgggataac
allose-6-phosphate isomerase/ribose-5-phosphate isomerase B ribose 5-phosphate isomerase B [EC:5.3.1.6]	rpiB	F<br>R	gtactggcgtcggtatttcg<br>accactcgtgaaccaaaagc
transaldolase B transaldolase [EC:2.2.1.2]	talB	F<br>R	gggcgtggtttctgtatctg<br>agaatttcgccgatgttacg
transaldolase A [EC:2.2.1.2]	talA	F<br>R	agttgatgcacgcctctctt<br>atttctcaacgccttgttgc

	ribokinase [EC:2.7.1.15]	rbsK	F	aaagctcaccggtattcgtg
			R	ccattcacgctagcccatac
	putative mutase YhfW phosphopentomutase [EC:5.4.2.7]	yhfW	F	cgcgatttgtggtgttagtg
			R	aactggctcaggatgtgacc
	phosphopentomutase [EC:5.4.2.7]	deoB	F	attgcctgccatgaagaaac
			R	tggaagttaccggctttgtc
	ribose-phosphate pyrophosphokinase [EC:2.7.6.1]	prs	F	ttgtggtttctccggacatc
			R	catcacctgtgaaacgttcg
	ribose 1,5-bisphosphokinase [EC:2.7.4.23]	phnN	F	aggattgccatacgctcaac
			R	caggcatggtgtttctcctt
	deoxyribose-phosphate aldolase [EC:4.1.2.4]	deoC	F	cggtaaccaacttcccacac
			R	caggtcaaaaccaacctgct
Fatty acid biosynthesis	acetyl-CoA carboxyltransferase subunit alpha<br>acetyl-CoA carboxylase carboxyl transferase subunit alpha [EC:6.4.1.2; 2.1.3.15]	accA	F	ccgtcaggatgagaaactgg
			R	atcggcgaagattttacgtg
	acetyl-CoA carboxyltransferase subunit beta<br>acetyl-CoA carboxylase carboxyl transferase subunit beta [EC:6.4.1.2; 2.1.3.15]	accD	F	aagcactgatgtcgctgatg
			R	atggcgaaacttgcagaaac
	biotin carboxyl carrier protein<br>acetyl-CoA carboxylase biotin carboxyl carrier protein	accB	F	aaagtcaacgtgggcgatac
			R	ctttcacggtaccggatttg
	biotin carboxylase<br>acetyl-CoA carboxylase, biotin carboxylase subunit [EC:6.4.1.2; 6.3.4.14]	accC	F	tatctgaacatcccggcaat
			R	tcgggccaatgaagataaag
	[acyl-carrier-protein] S-malonyltransferase [EC:2.3.1.39]	fabD	F	tattgcgaaagcgtgtgaag
			R	cgctcaaccgcttctttatg
	3-oxoacyl-[acyl-carrier-protein] synthase III [EC:2.3.1.180]	fabH	F	cgcagcttgtcagattcaaa
			R	aaccgacgaccagagcatac
	3-oxoacyl-[acyl-carrier-protein] synthase I [EC:2.3.1.41]	fabB	F	ctctgatggtgcagacatgg
			R	cgtgggagttcaggtaatcg
		fabF	F	aactcgtcggctttggtatg

	3-oxoacyl-[acyl-carrier-protein] synthase II [EC:2.3.1.179]		R	cgcgttaacgtagccaatct
	3-oxoacyl-[acyl-carrier protein] reductase [EC:1.1.1.100]	fabG	F	ccgtctgtcaaaagcggtaa
			R	tttcccatggtaccaaccac
	beta-hydroxyacyl-acyl carrier protein dehydratase/isomerase 3-hydroxyacyl-[acyl-carrier protein] dehydratase / trans-2-decenoyl-[acyl-carrier protein] isomerase [EC:4.2.1.59; 5.3.3.14]	fabA	F	cgaacatgctgatgatggac
			R	cataaccggatcgccaataa
	3-hydroxyacyl-[acyl-carrier-protein] dehydratase [EC:4.2.1.59]	fabZ	F	ccgttcttactggtggatcg
			R	ccattgcttccagaatcagc
	enoyl-[acyl-carrier protein] reductase I [EC:1.3.1.9; 1.3.1.10]	fabI	F	gtgcgatgttgcagaagatg
			R	gccaggtgcaaaaccaatag
	long-chain-fatty-acid--CoA ligase long-chain acyl-CoA synthetase [EC:6.2.1.3]	fadD	F	aggcacggtagtcaatttcg
			R	gggtttgacgtactgcatcc
Fatty acid degradation	acyl-CoA dehydrogenase [EC:1.3.99.-]	fadE	F	cgctgattacctacggcatt
			R	ccggtaatatccatcgcatc
	putative 2,3-dehydroadipyl-CoA hydratase enoyl-CoA hydratase [EC:4.2.1.17]	paaF	F	atcgttagccagcaaaatgg
			R	tgccagctgtaaggcgtatt
	3-hydroxyacyl-CoA dehydrogenase FadJ 3-hydroxyacyl-CoA dehydrogenase / enoyl-CoA hydratase / 3-hydroxybutyryl-CoA epimerase [EC:1.1.1.35; 4.2.1.17; 5.1.2.3]	fadJ	F	tctgataggcgtcagcacag
			R	agaatggagtgcggaacaac
	3-hydroxyacyl-CoA dehydrogenase / enoyl-CoA hydratase / 3-hydroxybutyryl-CoA epimerase / enoyl-CoA isomerase [EC:1.1.1.35; 4.2.1.17; 5.1.2.3; 5.3.3.8]	fadB	F	tctggaggaaggcattatcg
			R	tcgaggtattttgcgctacc
	3-ketoacyl-CoA thiolase FadI acetyl-CoA acyltransferase [EC:2.3.1.16]	fadI	F	cttaatgcccgtaccacctg
			R	taggttttcgccatttgctc
	3-ketoacyl-CoA thiolase acetyl-CoA acyltransferase [EC:2.3.1.16]	fadA	F	gtcattgtcgatgcaattcg
			R	cgcattaaatgagcggagag
	acetyl-CoA acetyltransferase acetyl-CoA C-acetyltransferase [EC:2.3.1.9]	atoB	F	atcgtcttggagacggacag
			R	aatgtagcgccagttcatcc
	putative acyltransferase acetyl-CoA C-acetyltransferase [EC:2.3.1.9]	yqeF	F	tcagttgcaggatgcttacg
			R	aacgtctgcccgttactttg
	fused 2-acylglycerophospho-ethanolamine acyltransferase/acyl-acyl carrier protein	aas	F	aaaacgattgccgacttcac

	synthetase acyl-[acyl-carrier-protein]-phospholipid O-acyltransferase / long-chain-fatty-acid--[acyl-carrier-protein] ligase [EC:2.3.1.40 6.2.1.20]		R	tctgcacctgtaagcagtg g
	putative 3-phenylpropionate/cinnamate dioxygenase ferredoxin reductase subunit 3-phenylpropionate/trans-cinnamate dioxygenase ferredoxin reductase component [EC:1.18.1.3]	hcaD	F	atgaacgacctccactctc g
			R	ttacaccggaatgcagatg a
Glycerolipid metabolism	glycerate 2-kinase 2 glycerate 2-kinase [EC:2.7.1.165]	glxK	F	ggcgaattgccatattgaa g
			R	taattttgcagcccctgttc
	glycerate 2-kinase 1 glycerate 2-kinase [EC:2.7.1.165]	garK	F	gaaggtaccgattggtgtc g
			R	tgctggtcaatacgctgaa g
	aldehyde reductase, NADPH-dependent alcohol dehydrogenase (NADP+) [EC:1.1.1.2]	yahK	F	agccgttggtgatcaggta g
			R	acccgtcttcacactcttcg
	dihydroxyacetone kinase subunit M phosphoenolpyruvate---glycerone phosphotransferase subunit DhaM [EC:2.7.1.121]	dhaM	F	cggtacaggcaaaatcaa cc
			R	ggcttctgctttcgctgtta
	L-1,2-propanediol dehydrogenase/glycerol dehydrogenase glycerol dehydrogenase [EC:1.1.1.6]	gldA	F	aaatgagatcgaccgtctg c
			R	taccggaacacccatgaa at
	glycerol kinase [EC:2.7.1.30]	glpK	F	ttgctgtttggtacggttga
			R	agcattttgtcgtcccagtc
	putative phosphate acyltransferase phosphate acyltransferase [EC:2.3.1.274]	plsX	F	tttgccattatgggctcagt
			R	tcccgaatactgtcgagac c
	putative glycerol-3-phosphate acyltransferase acyl phosphate:glycerol-3-phosphate acyltransferase [EC:2.3.1.275]	plsY	F	gttacgtatcggtggcaag g
			R	cagaaggggctgacacct aa
	glycerol-3-phosphate 1-O-acyltransferase glycerol-3-phosphate O-acyltransferase [EC:2.3.1.15]	plsB	F	tactgcgctactggcatca c
			R	tggctgaaggaacggtag ag
	1-acylglycerol-3-phosphate O-acyltransferase PlsC 1-acyl-sn-glycerol-3-phosphate acyltransferase [EC:2.3.1.51]	plsC	F	tggtgacagcatcgaacat c
			R	accagtacaactgcccga ag
	diacylglycerol kinase (ATP) [EC:2.7.1.107]	dgkA	F	tcaaagctgctggctattcc
			R	atgaccaccgccaacaata c
	alpha-galactosidase [EC:3.2.1.22]	melA	F	cgcatattgtggtgcgtaa g
			R	tcaccgtgcaaggttcata a

Housekeeping gene	16S ribosomal RNA	rrsA	F	attgacgttacccgcagaag
			R	attccgattaacgcttgcac
	integration host factor subunit beta	ihfB	F	tttcggcagtttctctttgc
			R	atcgcgcagttctttaccag
	uroporphyrin-III C-methyltransferase	cysG	F	gtttatttttggccgtggtg
			R	gatgcgtgagtggaatacc
	MFS transporter	hcaT	F	ctgatgctggtgatgattgg
			R	agtcgcactttgccgtaatc
	L-idonate/5-ketogluconate/gluconate transporter	idnT	F	aagtgggttttgtcctgctg
			R	acagagagcgctgctaccat
	outer membrane protein A	ompA	F	tgagtacgcgatcactcctg
			R	ctgaccgaaacggtaggaaa
	RNA polymerase, sigma 70 (sigma D) factor	rpoD	F	ttcgtacgcaagaacgtctg
			R	aggtatcgctggtttcgttg
